# Text Mining of CVD Synthesis Recipes for 2D Materials

**DOI:** 10.1002/adma.202509132

**Published:** 2026-03-12

**Authors:** Ang‐Yu Lu, Richard A. Chen, Aijia Yao, Meng‐Chi Chen, Ji‐Hoon Park, Tianyi Zhang, Xudong Zheng, Nannan Mao, Jiangtao Wang, Zhien Wang, Tomás Palacios, Jing Kong

**Affiliations:** ^1^ Department of Electrical Engineering and Computer Science Massachusetts Institute of Technology Cambridge Massachusetts USA; ^2^ Department of Civil and Environmental Engineering Massachusetts Institute of Technology Cambridge Massachusetts USA; ^3^ Department of Materials Science and Engineering Massachusetts Institute of Technology Cambridge Massachusetts USA

**Keywords:** 2D materials, chemical vapor deposition, machine learning, natural language processing, text mining

## Abstract

A vast amount of scientific knowledge is embedded in journal articles as unstructured text, creating challenges for efficiently extracting detailed insights. Traditionally, expert‐authored reviews summarize research progress, but they often struggle to capture the intricate synthesis protocols in individual papers and provide limited quantitative comparisons of experimental techniques. Recent advancements in machine learning, particularly natural language processing (NLP), have enabled automated text mining and information extraction. However, in materials science, most approaches have focused on refining model architectures rather than addressing domain‐specific challenges such as data annotation and the extraction of complex synthesis details. We present a machine learning framework for extracting synthesis protocols of 2D materials, including graphene and TMDs, from publications spanning 1980–2022. By combining named entity recognition (NER) and extractive question answering (EQA), we retrieve both categorical and numerical synthesis parameters. Generative models are further used to summarize and generate experimental recipes, enabling knowledge transfer across material systems. Our domain‐specific, fine‐tuned models offer improved precision and interpretability compared to general‐purpose approaches. This scalable framework helps unlock hidden insights from literature, supporting data‐driven synthesis optimization and accelerating materials discovery.

## Graphical abstract

A lightweight, multi‐stage natural language processing framework utilizes fine‐tuned BERT models to extract chemical vapor deposition synthesis knowledge from diverse 2D materials literature. The domain‐adapted workflow integrates classification, named entity recognition, and extractive question answering to systematically retrieve categorical and numerical synthesis parameters. Generative models further summarize experimental recipes, enabling transparent, large‐scale mining and fostering cross‐material insights in materials science.

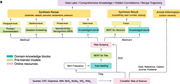

## 1 Introduction

2D materials, such as graphene and transition metal dichalcogenides (TMDs), have garnered significant attention in recent years due to their unique physical, electronic, and optical properties. These materials offer exceptional potential for applications in nanoelectronics, optoelectronics, and energy storage. Among the various synthesis techniques, chemical vapor deposition (CVD) has emerged as a key method for fabricating high‐quality, large‐area 2D materials, enabling precise control over their properties. However, challenges remain in controlling factors such as layer thickness and minimizing defects, which are critical for the performance and scalability of devices based on 2D materials.

Machine learning is accelerating discovery and synthesis optimization in 2D materials, especially through integration with high‐throughput experiments and computational modeling. Recent deep learning pipelines for automated 2D material identification [1] exemplify ML's potential for efficient characterization, paving the way for more scalable, data‐driven research.

The amount of research in 2D materials and volume of published literature has exploded, making it difficult to manually track and extract valuable synthesis details. Traditionally, 2D materials research progress has been comparatively summarized through expert‐authored review articles, but this approach is often limited in scope, especially when it comes to extracting detailed experimental protocols and quantitatively comparing synthesis techniques. In this context, advancements in machine learning, particularly natural language processing (NLP), offer an opportunity to automate the extraction of scientific knowledge from unstructured text.

Efforts to develop machine learning models [2, 3] for extracting information from scientific literature have primarily focused on either word‐embedding methods or context‐aware models. Word‐embedding techniques, such as bag‐of‐words [4] and Latent Dirichlet Allocation (LDA) [5], are unsupervised methods that identify relationships between terms and group them into topics. While these models are computationally efficient, they struggle to capture long‐range dependencies and provide a limited view of the full context of research articles [6].

On the other hand, supervised context‐aware models, such as recurrent neural networks (RNNs) and transformers like BERT (bidirectional encoder representations from transformers) [7], have demonstrated superior performance in extracting structured information, such as synthesis recipes from scientific publications. Transformer‐based large language models (LLMs), including more recent variants like GPT [8], Gemini [9], and Llama [10] are highly capable of understanding comprehensive information. However, LLMs require substantial computational resources, large datasets, and high‐quality annotations from domain experts, making them time‐consuming and resource‐intensive to train and utilize for predictions. Moreover, current models often focus on extracting general material knowledge, without delving into the underlying physical mechanisms, which limits their applicability.

In this work, we utilize lightweight pre‐trained models like BERT to efficiently extract synthesis knowledge for 2D materials, including graphene and transition metal dichalcogenides (TMDs), from scientific literature. BERT's lightweight size is demonstrated by its model size of 110 M parameters, compared to typical LLM model sizes of several billion parameters. BERT's computational efficiency, accessible last layer hidden state outputs, and ease of fine‐tuning with smaller datasets make it well‐suited for tasks such as classification, named entity recognition (NER), and extractive question‐answering (EQA). We leverage these strengths and fine‐tune separate BERT models for classification of synthesis results from abstracts, named entity recognition (NER) for extracting all mentions of categorical and numerical entities, and extractive question answering (EQA) for extracting recipe categorical and numerical data, providing more precise and comprehensive information than NER alone. The stepwise nature of our framework allows us to independently inspect model outputs after each step and mitigate model prediction errors through further finetuning or rule‐based corrections, in contrast to the low interpretability and risk of hallucination of LLMs [11].

Beyond these tasks, we apply GPT‐Neo as the generative model for summarization and prompting, synthesizing experimental recipes by aggregating and analyzing data from multiple documents. This approach enables us to identify recurring patterns and relationships in synthesis protocols across different materials and time periods. Our framework, using classification, NER, and EQA models, effectively extracts both categorical and numerical data from 2D material synthesis publications, tracing synthesis trajectories and enabling comparisons between methods. Generative models then enhance our ability to summarize and connect knowledge, offering a comprehensive approach to automate data extraction and provide deeper insights into material synthesis, benefiting the broader scientific community. To further ensure transparency and rigor, we explicitly describe benchmarking and comparative evaluation procedures throughout the manuscript so that readers are clearly informed about model comparisons at each stage of the pipeline. Overall, we present a domain‐adapted, multi‐stage workflow for extracting complex CVD synthesis information from scientific literature. Beyond data extraction, our framework generates new materials science insights by revealing emergent synthesis patterns across thousands of experiments. Through large‐scale data synthesis, we uncover a constant‐dose kinetic principle governing CVD growth, reconstruct the temporal evolution of TMD synthesis strategies, and demonstrate knowledge transfer capabilities through learned chemical relationships that suggest novel precursor combinations for under‐explored materials.

## 2 Overview and Title Classification

**FIGURE 1:**
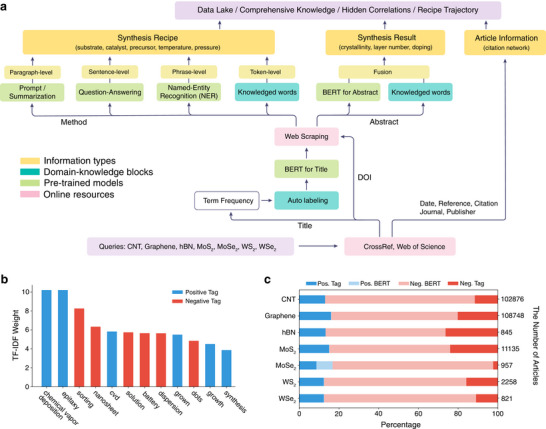
Overview of the framework. (a) Schematic of the flowchart for text mining for 2D materials. (b) TF‐IDF weights for each of the tokens. The positive tag denotes CVD‐related terms for 2D material synthesis, while the negative tag indicates non‐CVD‐related terms. (c) the percentage of results using positive and negative keywords and BERT search for titles.

Incorporating domain knowledge into transformer‐based language models, such as BERT, enhances the accuracy and efficiency of the training process. Figure 1a outlines the text mining process, consisting of three primary components: online resources, domain knowledge, and pre‐trained models. First, we input 2D materials, including chemical formula and abbreviations, as queries for publisher databases, such as Web of Science (WoS) and CrossRef, retrieving article information such as citations, titles, and publication dates. We first filter out articles that are unrelated to CVD through a term‐based search and prediction with a fine‐tuned BERT model. We find identifying keywords indicative of titles related to CVD and titles unrelated to CVD by applying domain knowledge in CVD synthesis for 2D materials, calculating term frequencies for titles, and ranking terms accordingly in Figure 1b. For example, terms such as chemical vapor deposition, epitaxial, and CVD pertain to CVD‐related concepts, while solution, nanosheets, and sorting typically refer to unrelated chemically exfoliated 2D materials or solution‐based carbon nanotubes. We also fine‐tune a pre‐trained BERT model, achieving a high macro F1 score of 0.92 in classifying CVD‐related titles. We first classify articles with identifying keywords. Then, among titles without identifying keywords, we employ the fine‐tuned BERT model to predict titles, as depicted in Figure 1c. For articles related to CVD we obtain article content through web scraping or publisher sources and identify abstracts and method sections based on HTML elements. After acquiring content from publishers, we extract growth results from abstracts and growth recipes from methods. In abstracts, we extract growth outcomes for 2D material synthesis, such as monolayer, bilayer, and multilayer (single‐walled, double‐walled, and multi‐walled for carbon nanotubes), as well as plasma, using a BERT classifier combined with keyword tags. To extract growth recipes from method sections, we adopt four distinct strategies. First, we apply a token‐level rule‐based dictionary to locate keywords. Second, we perform phrase‐level NER to pinpoint parameter tokens. Third, we employ an extractive question‐answering (EQA) model to procure context‐aware entity answers for recipes. Finally, we implement paragraph‐level prompts to generate growth recipes for novel 2D materials, enabling knowledge transfer between materials.

Beyond its primary extraction capabilities, our framework creates a database that lays the groundwork for future scientific tools. This curated knowledge base could serve as a foundational source for future Retrieval‐Augmented Generation (RAG) systems, aiming to ground language models in validated literature to enable evidence‐based hypothesis generation. The extracted recipes also serve as valuable few‐shot learning examples.

## 3 Abstract Classification and Named Entity Recognition (NER)

Preparing high‐quality expert‐labeled data is a critical and time‐consuming task for training deep neural networks. In this study, we present an empirically efficient method that leverages domain knowledge to pre‐annotate data, which expedites the annotation process for experts. The architecture of abstract classification is illustrated in Figure S1. For instance, monolayer and single‐layer indicate 2D monolayer synthesis, while multilayer and few‐layer signify 2D multilayer synthesis. We fine‐tune a multi‐class model that integrates a pre‐trained BERT model with keyword tags to identify results for abstracts. The confusion matrix of the abstract BERT classifier demonstrates a higher weighted F1 score of 0.88, as shown in Figure S3, compared to the standalone BERT classifier with a weighted F1 score of 0.8. For extracting growth recipes, we first pre‐annotate parameters using a dictionary containing keywords and units, followed by corrections made by CVD experts. The NER model is then trained based on the annotated data, yielding a high macro F1 score of 0.92, according to the confusion matrix in Figure S5. Our dataset extracted by abstract classification and NER enables visualization of correlations between results and recipes, catalysts and 2D materials, and the popularity of material synthesis methods over time. We evaluate the importance of characterization tools for 2D materials by examining the ratio of citations to publications, as shown in Figure 2a. Raman spectroscopy emerged as the most popular tool for 2D materials, attributed to its fast and non‐destructive nature [12, 13]. Atomic force microscopy (AFM) has the highest citation‐to‐publication ratio, attributed to its rapid, non‐destructive nature and widespread availability. In addition, among all electron‐based measurements (indicated in orange), scanning transmission electron microscopy (STEM) exhibits a higher citation‐to‐publication ratio as it provides atomic lattice information that is crucial for understanding 2D material properties such as strain, defects, and dopants [14, 15]. Conversely, X‐ray diffraction analysis has a lower citation‐to‐publication ratio due to its lower signal intensities for the thin atomic thickness of 2D materials, making it less suitable for characterizing 2D materials. When weighting publications by number of citations, we can rediscover potential substrate and catalyst combinations for bilayer graphene growth, such as methane‐nickel and acetylene‐copper, compared to the highly published methane‐copper combination, as shown in Figure 2b. Through the time trajectories of catalyst usage, we observe knowledge transfer between materials like graphene and carbon nanotubes. As depicted in Figure 2c, methane is the most widely used precursor for both monolayer and multilayer graphene growth. Meanwhile, acetylene and ethylene are promising precursors for multilayer graphene growth, possibly due to their lower decomposition temperature compared to methane, which substantially increases the carbon concentration during graphene growth. Figure 2d shows how 2D materials researchers transferred their insights from CNT synthesis to graphene synthesis. Researchers initially employed nickel as the substrate and subsequently shifted to copper. Copper also emerged as the better choice for graphene synthesis due to its low carbon solubility, which prevents multilayer graphene precipitation during the cooling process. We believe similar knowledge transfers of growth parameter selection can occur between other 2D materials.

**FIGURE 2:**
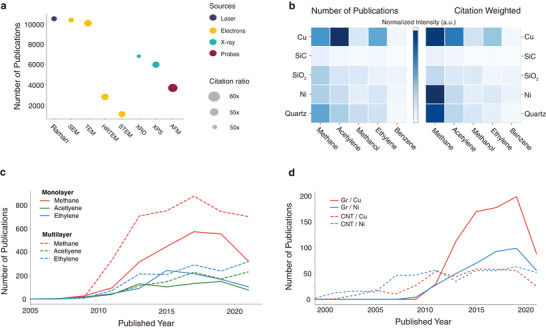
Categorical parameters extracted by NER. (a) The number of publications using characterization tools. The coded colors indicate the sources of tools, and the sizes of the circles represent the citation ratio (Raman: Raman spectroscopy, SEM: scanning electron microscopy, TEM: transmission electron microscopy, HRTEM: high‐resolution transmission electron microscopy, STEM: scanning transmission electron microscopy, XRD: X‐ray diffraction, XPS: X‐ray photoelectron spectroscopy, AFM: atomic force microscopy). (b) The number of publications of precursor‐substrate combinations for bilayer graphene growth with unweighted (left‐hand side) and weighted by number of citations (right‐hand side). (c) the trajectories of precursor gasses for monolayer (the solid lines) and multilayer (dashed lines) graphene growth from 2000 to 2022. (d) the trajectories of catalysts for graphene (the solid lines) and carbon nanotube (dashed lines) growth from 2000 to 2022. The shift from nickel to copper for graphene reflects improved control over synthesis, with copper enabling monolayer graphene growth. These trends highlight the evolving understanding of catalyst selection for graphene synthesis.

## 4 Extractive Question Answering (EQA)

**FIGURE 3:**
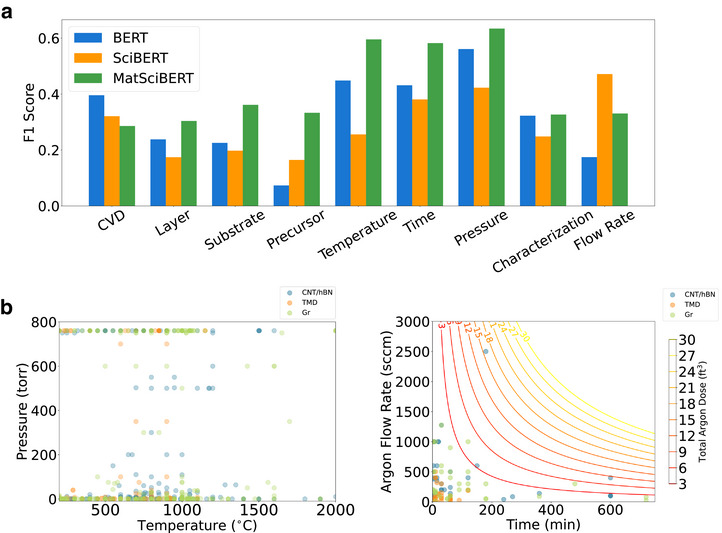
Performance and predictions from extractive question‐answering models. (a) F1 score performance of BERT, SciBERT, and MatSciBERT QA models for nine parameters compared to manual annotations. (b) A scatter plot illustrating temperature versus pressure combinations for the 5000 top‐cited 2D materials papers acquired via web scraping, categorized by material group. We exclude predictions with temperature outside of 200–2000°C or pressure exceeding 760 torr and assume they are errors. (c) A scatter plot displaying time versus argon flow rate combinations for the 5000 top‐cited 2D materials papers, categorized by 2D material type. Contour lines indicating total dose of argon, calculated as growth time times argon flow rate, are overlaid on the scatter plot.

Although NER is a widely used approach for information extraction from literature, it encounters challenges in identifying relationships between entities in a sentence or paragraph. As a result, NER predictions may struggle to differentiate between various parameter values associated with distinct objects. For example, numerous temperature values correspond to different experimental components, such as the precursor and substrate. To identify the contextual background associated with each parameter, we performed an extractive question answering (EQA) task by employing three different BERT variants, extracting parameters using handcrafted questions for each of nine entities listed in Table S1. Our approach resembles that of QaNER, which demonstrates high performance on entity extraction tasks by inputting handcrafted questions into a QA model. We developed a domain‐specific question answering model by adding a question answering head to MatSciBERT [16], a BERT variant trained on materials science literature, and fine‐tuning the model using the SQuAD1.1 dataset. We compared the MatSciBERT model with a BERT question answering model fine‐tuned on SQuAD1.1, as well as a question answering model developed by Otegi et al. using SciBERT and fine‐tuning on SQuAD2.0 and QuAC [17]. We evaluate the macro‐averaged F1 score on manually annotated 2D materials articles by counting the number of overlapping words between prediction and truth and refer the reader to Section S1.4.3 for the full computation details. Figure 3a shows that MatSciBERT outperformed the other models in retrieving information related to layer, substrate, precursor, temperature, time, pressure, and characterization entities, while BERT performed highest for the CVD entity and SciBERT had a higher F1 score for extracting flow rates. We hypothesize that BERT's higher performance on the CVD entity is due to the simplicity of the task, because the gold answer was most often “chemical vapor deposition.” We hypothesize that SciBERT's higher performance on the flow rate entity is due to the longer nonzero gold (ground truth) answers, as shown in Table S2. Figure S8 shows that SciBERT exhibits longer prediction lengths and a higher overall R^2^ score with gold answer lengths than BERT and MatSciBERT. Arantxa et al. attribute the long responses of the SciBERT QA model to its fine‐tuning on the QuAC dataset [18]. Therefore, SciBERT's lengthy answers best match the lengthy flow rate gold answers. We use the best‐performing model for each parameter to extract the answers for the most accurate predictions.

We perform a consistency check of EQA predictions using term frequency‐inverse document frequency (TF‐IDF). Figure S9 shows the top 10 TF‐IDF ranked terms for each named entity, confirming that the top ranked terms from EQA predictions agree with our domain knowledge of the most important terms within each entity. TF‐IDF also identifies shortcomings of the BERT, SciBERT, and MatSciBERT models. The BERT‐based models misclassify some carrier gases as precursors, “degc” as a unit for time, pressure, and flow rate, and the common pressure of 760 as a time entity.

Finally, we employ regular expressions to extract numerical values from the answers generated by EQA for the 5000 top‐cited 2D materials articles. In Figure 3b, we plot the temperature versus pressure for 2D materials, illustrating that most efforts are focused on either ambient pressure (≈760 torr) or near the base pressure of pumps (≈1 torr). Furthermore, CNT/hBN is the most common material category for experiments with an intermediate pressure (>1 torr and <760 torr), suggesting potential for knowledge transfer from CNT/hBN experiments to TMD and Gr experiments using intermediate pressure. The 50–200 Torr gap for TMDs suggests an underexplored regime that could combine APCVD precursor availability with LPCVD uniformity while avoiding turbulence and vacancy formation [19, 20, 21]. When compared with the pressure entity results from the NER method in Figure S6, the findings from the EQA method seem more plausible for numerical answers, as a pressure control system is more expensive than ambient pressure or pump base pressure. Figure 3c shows a scatter plot of growth time versus argon gas flow rate, along with contour lines for total dose of argon gas, which represents the approximate total gas volume flowed over the substrate. We calculate the total dose of argon gas by multiplying growth time and flow rate. Figure 3c shows alignment of growth time and flow rate combinations along contour lines, suggesting that a specific range of total gas exposure leads to successful materials growth. Figure S10 shows similar scatter and contour plots for acetylene, helium, hydrogen, methane, and nitrogen, reinforcing the observation that similar total gas doses favorably influence CVD kinetics. Finally, we examine the interaction between flow rate and tube diameter. Figure S11 shows that smaller tube diameters around 1″ are more common. When we normalize by tube cross sectional area to compute velocity, we observe that velocities are higher for smaller tube diameters as expected.

## 5 Generative Models

**FIGURE 4:**
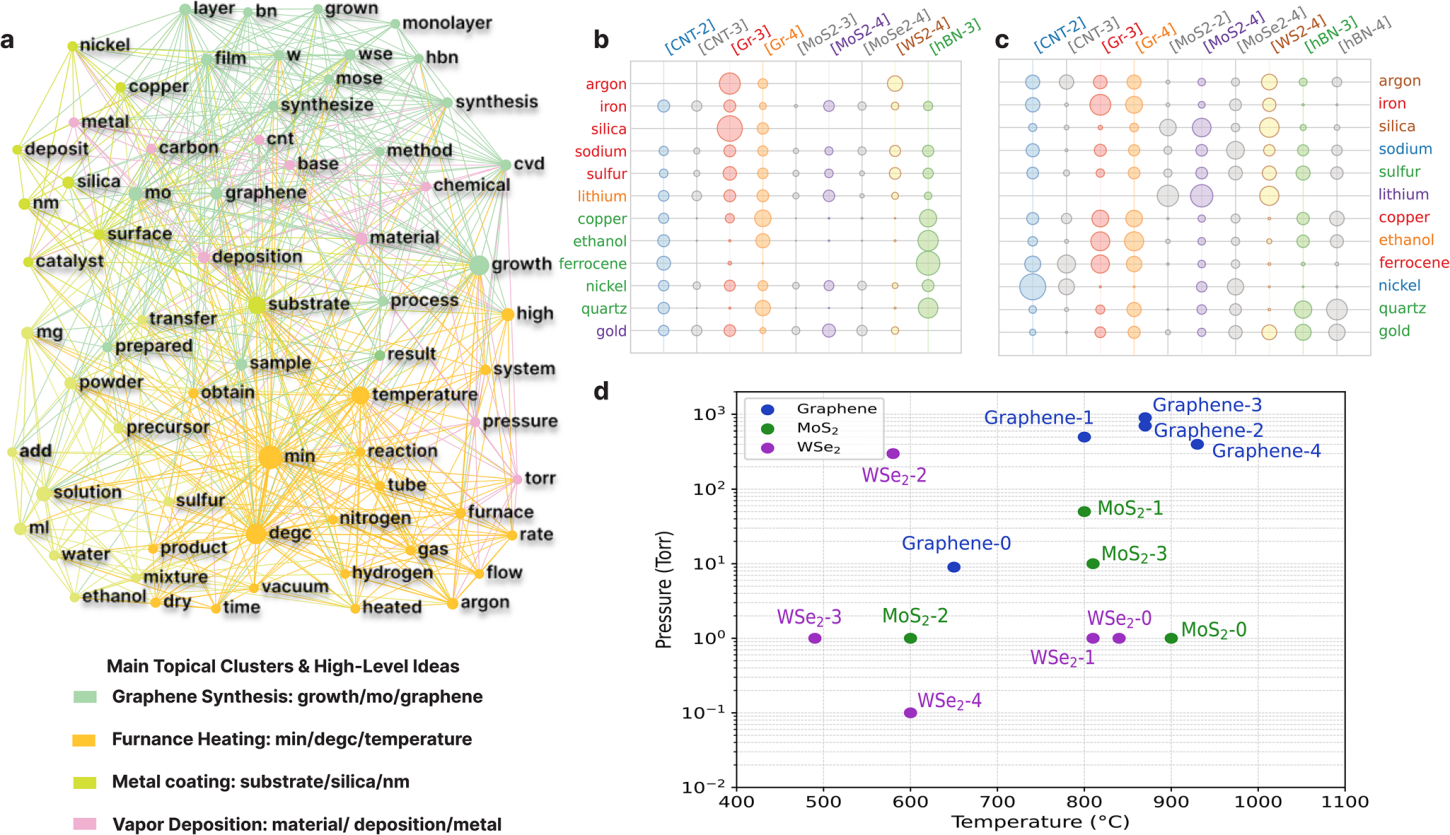
Generative models analytics. (a) Top four topical clusters grouped by nearest neighbor based on keywords in generated summaries. (b, c) Termite plots for the normalized co‐occurrence of recipe keywords and group label in prompt‐generated outputs under randomness 0.5 and 1, where the bubble size indicates the frequency of co‐occurrence in the prompt‐generated text output for corresponding entries. (d) Temperature–pressure scatter plot of generated synthesis conditions with reasonable operating ranges shown in a colored gradient.

To further investigate correlations and trends among the recipes for different materials reported in published literature, we demonstrate two approaches using generative models: multi‐document summarization and prompt‐based text generation. First, we employ an unsupervised multi‐document summarization model to generate summaries for experiment sections, leveraging the common written structures shared by the recipes. By categorizing the literature in our dataset according to the synthesis materials, we obtained summaries of reported synthesis recipes for 7 distinct materials (see the details in Section S1.5). The generated summaries function effectively as raw input for lightweight text mining methods such as TF‐IDF, latent Dirichlet allocation (LDA), and text network analysis (TNA). The LDA model, based on term frequency calculation, clusters the summaries into multiple groups, potentially corresponding to subtopics in recipes, as shown in Figure S12. To determine the topics of these groups, we input the summaries into the TNA model, generating the network depicted in Figure 4a. The connections between recipe keywords indicate correlations among various synthesis terms. For example, CNT and carbon are positioned in close proximity within the network, even though they belong to distinct LDA topics. More informative connections between keywords can be easily identified within the network, such as the association of “graphene” with specific growth conditions, or the link between “CNT” and particular catalyst materials. These connections show the underlying pattern between certain keywords and synthesis methods.

As an alternative approach, we fine‐tuned a prompt‐based text generation model to generate recipes with a holistic view among low‐dimensional materials without losing details during summarization. We designed prompts to generate recipes, focusing on catalysts, substrates, temperatures, pressures, characterizations, and applications. In generative models, text flow control can be achieved by selecting appropriate generation settings, such as beamwidth and randomness. Termite plots demonstrate the co‐occurrence of keywords in the prompting output with different randomness generative settings for each material‐year group, as illustrated in Figure 4b,c and explained in Section S1.5. The usage trend of the same recipe component can be exemplified by the increased use of copper from 2010 to 2016 for graphene synthesis, shown by comparing the bubble size of “copper.” Another example is “ferrocene,” which was primarily predicted to be used in CNT and hBN synthesis with medium randomness. When the randomness increases to 1, the termite plot suggests ferrocene could be the catalyst for graphene growth, implying potential knowledge transfer among different materials and potential creativity in this model for future exploration of novel materials.

Lastly, the readability of all generated text can be assessed by applying a Kincaid test to each of the outputs, with the distribution presented in Figure S15. The summaries yield an average of 70.9 (fairly easy to read, equivalent to the 7th grade in the US), demonstrating improved readability compared to the input text and prompted outputs (on average). This finding reveals a potential trade‐off between readability and details for generated text, particularly for synthesis recipes. To further enhance the reliability of the generated recipes, we transitioned from GPT‐Neo to Llama‐2‐7b‐hf for prompt‐based generation, while retaining our structured data‐labeling approach. This robust model not only maintains the readability of outputs but more consistently produces chemically plausible parameters. Figure 4d confirms that the predicted recipes cluster within reasonable temperature–pressure windows for all material categories, supporting the use of our framework as a decision‐support tool for realistic synthesis design.

## 6 Summary

Understanding academic publications is an essential step in guiding scientific researchers toward the synthesis of tailored materials. In this work, we demonstrate a framework for extracting experimental recipes from literature to gain a comprehensive understanding of 2D material synthesis by monitoring, comparing, and evaluating the synthesis parameters across different materials and growth outcomes. We employed NER and EQA models to extract experimental recipes for both categorical and numerical parameters, as well as conducted statistical analysis to gain profound insights into the growth mechanisms. Furthermore, we used generative models to summarize and generate experimental recipes, providing a broad understanding of 2D material growth from multi‐document inputs. This approach not only identifies patterns and trends in synthesis protocols but also uncovers opportunities for knowledge transfer, such as applying successful graphene growth techniques to transition metal dichalcogenides (TMDs).

Nevertheless, this work is only an initial exploration and still very limited in its utility and impact. Several near‐term improvements can be seen. Firstly, fine‐tuning a masked language model for domain adaptation in 2D material articles could enhance the performance of downstream tasks. Secondly, integrating various downstream tasks may improve recipe extraction performance. For instance, applying a prompt‐based learning QA method combined with a NER model could enable the extraction of more information from literature, while a two‐stage entity span recognition and classification approach similar to Split‐NER could improve our QA model performance. While our current efforts focus on refining model architecture and performance for 2D material knowledge, we anticipate that this work may serve as a foundation for using natural language processing to investigate other existing materials and discover new materials in the future.

## Author Contributions

A.‐Y.L. and J.K. conceived and designed the experiments. A.‐Y.L., R.A.C., and A.Y. collected and performed all the data analysis and machine learning models. M.‐C.C., J.‐H.P., T.Z., X.Z., N.M., J.W., and Z.W. annotated the training data. J.K. supervised this work. A.‐Y.L., R.A.C., A.Y., M.‐C.C., and J.K. co‐wrote the paper with inputs from all the authors. All the co‐authors have reviewed the manuscript and provided their input.

## Conflicts of Interest

The authors declare no conflicts of interest.

## Supporting information




**Supporting File**: adma72743‐sup‐0001‐SuppMat.docx.

## Data Availability

The data that support the findings of this study are available from the corresponding author upon reasonable request.
