# Supplementary material for: Text Mining of CVD Synthesis Recipes for 2D Materials
Source: Adv Mater. 2026 Mar 12;38(20):e09132. doi: 10.1002/adma.202509132 (PMC13054107; doi:10.1002/adma.202509132)
Supplement: Supplementary file 1 — Supporting File: adma72743‐sup‐0001‐SuppMat.docx. [file ADMA-38-e09132-s001.docx]

Supplementary Information

1. Additional Method Description
   1. Data Preparations
      1. Article Information for 2D Materials

We utilized CrossRef's RESTful API and Web of Science to acquire information about articles, including title, DOI, publication date, references, citation count, journal, and publisher. We use the full names and abbreviations of 2D materials as search queries in literature databases, such as carbon nanotubes (CNT), graphene, hexagonal boron nitride (hBN), molybdenum disulfide (MoS_2_), molybdenum diselenide (MoSe_2_), tungsten disulfide (WS_2_), and tungsten diselenide (WSe_2_). Citation counts for each article were obtained using CrossRef's Habanero API, with the DOI of the articles as the query.

- - 1. Term Frequency and Auto-Labeling

The annotation process can be quite time-consuming during data preparation. To address this, we utilized our domain knowledge to automatically label titles for machine learning models. After retrieving titles from CrossRef and Web of Science, we tokenized the titles into words and calculated the total word frequency count, as illustrated in Figure 1b. Using our expertise in 2D material synthesis, we assigned CVD-related words, such as “chemical vapor deposition”, “epitaxy”, and “CVD”, as positive labels, and non-CVD-related words, such as “sorting”, “nanosheet”, and “solution”, as negative labels for the BERT-title classification model. Through the fine-tuning process, the BERT-title model can predict titles that don’t contain the keywords.

- - 1. Title Classification by the Fine-Tuned BERT Model

We fine-tuned a title classifier by employing a pre-trained BERT model combined with two fully connected layers, creating labels based on term frequencies and our domain knowledge without manual annotation to extract CVD-related titles. We set the hyperparameters to 30 training epochs and a learning rate of 5E-5, resulting in a macro-averaged F1 score of 0.98. Relying on the predictions from the model and the tags from the keyword dictionary, we retained the CVD-related titles for further refinement.

- - 1. Web Scraping for Content Extraction

We obtained the web content of articles in HTML/XML format from 16 different publishers with permission. Using BeautifulSoup, we developed a web scraping tool to extract text from published articles. First, we accessed the website via URL links corresponding to the DOI numbers of the articles. Then, we divided the full text of the articles into abstract and method sections based on their HTML/XML subtitles or paragraph content. All web scraping processes adhered to the policies of journal publishers. Moreover, we directly requested the content of articles published in American Chemical Society (ACS) journals from the publisher.

- 1. Abstract Classification
     1. Process Flow of Keyword Tagging

After retrieving each article’s content through web scraping, we first eliminate articles lacking either an abstract or method section. Utilizing domain knowledge in 2D materials, we create a keyword dictionary to tag our abstracts. For instance, we assign single-walled, double-walled, and multi-walled labels for carbon nanotube synthesis, and single-layer, double-layer, and multilayer labels for 2D material synthesis. Concurrently, we use radio-frequency, RF, and plasma as keywords for plasma-related articles. In cases where none of the keywords can be found in an abstract or title, we designate multi-walled and single-layer as default tags for CNT- and 2D-related articles, respectively. To evaluate the accuracy of the tagging method, we compare the automatically assigned labels with manually annotated data, which serves as the gold standard. The annotated dataset was curated by domain experts, ensuring high-quality reference labels for model evaluation. We then compute the F1 score using the Scikit-learn module with multilabel classification metrics, which accounts for cases where an article may have multiple relevant labels.

- - 1. Data Processing for the Abstract Classification

During the processing stage, we concatenated the title and abstracts as a combined input for classifying synthesis results using the BERT model. The dataset was then shuffled and split into 80% training and 20% validation data. To ensure accurate labeling for the multilabel classification task, we collaborated with domain experts who annotated the training data using the Label Studio platform. Since a single article can contain multiple synthesis results, experts assigned multiple labels such as "substrate," "catalyst," and "temperature" to capture the diverse experimental parameters described in each paper.

- - 1. The Model Architectures

To generate label predictions, we first process the combination of a title and abstract through a tokenizer to obtain individual tokens, then feed the tokenized text into a pre-trained BERT model, as illustrated in Figure S1. Following this, we pass the last hidden state layer into a linear layer. We combine the hidden state layer with keyword tags from Supplementary Section 1.2.1 through a fusion layer, the types of which are depicted in Figure S2:

1. Logits Only layer: We pass the last hidden state into an additional linear layer.
2. Pool layer: We combine the last hidden state layer and tags by taking the maximum at each point in the array and then passing that through a linear layer.
3. Add layer: We add the last hidden state layer and tags together and then pass through another linear layer.
4. Concatenate layer: We concatenate the logits and tags tensors, then run this through a linear layer that halves the tensor size.

The output from the fusion layer is then passed through a sigmoid layer and a BCELoss function for our multilabel classification with four labeled types: single-layer, bilayer, multilayer, and few-layer. Each predicted output ranges between 0 and 1 and is subsequently processed by a threshold, where values above the threshold cutoff of 0.4 are mapped to a 1 for the label, while values below are mapped to 0.

- - 1. Hyperparameters Settings

To optimize the hyperparameters, we adjusted the learning rate, number of epochs, batch size, threshold value, and warmup of the learning rate scheduler. We employed Bayesian Optimization to identify the ideal combination of these parameters. The Adam optimizer was used in conjunction with a 0.05 weight decay rate. For fine-tuning the model parameters, we explored various learning rate schedulers, including constant, linear, and cosine schedulers, each with the option to incorporate a warmup phase to gradually introduce the learning rate.

- - 1. Performances and Evaluation Metrics

To assess the performance of each model, we examined the weighted average F1 and ROC-AUC curve scores for various fusion layer types. The confusion matrix for the concatenate mode is depicted in Figure S3.


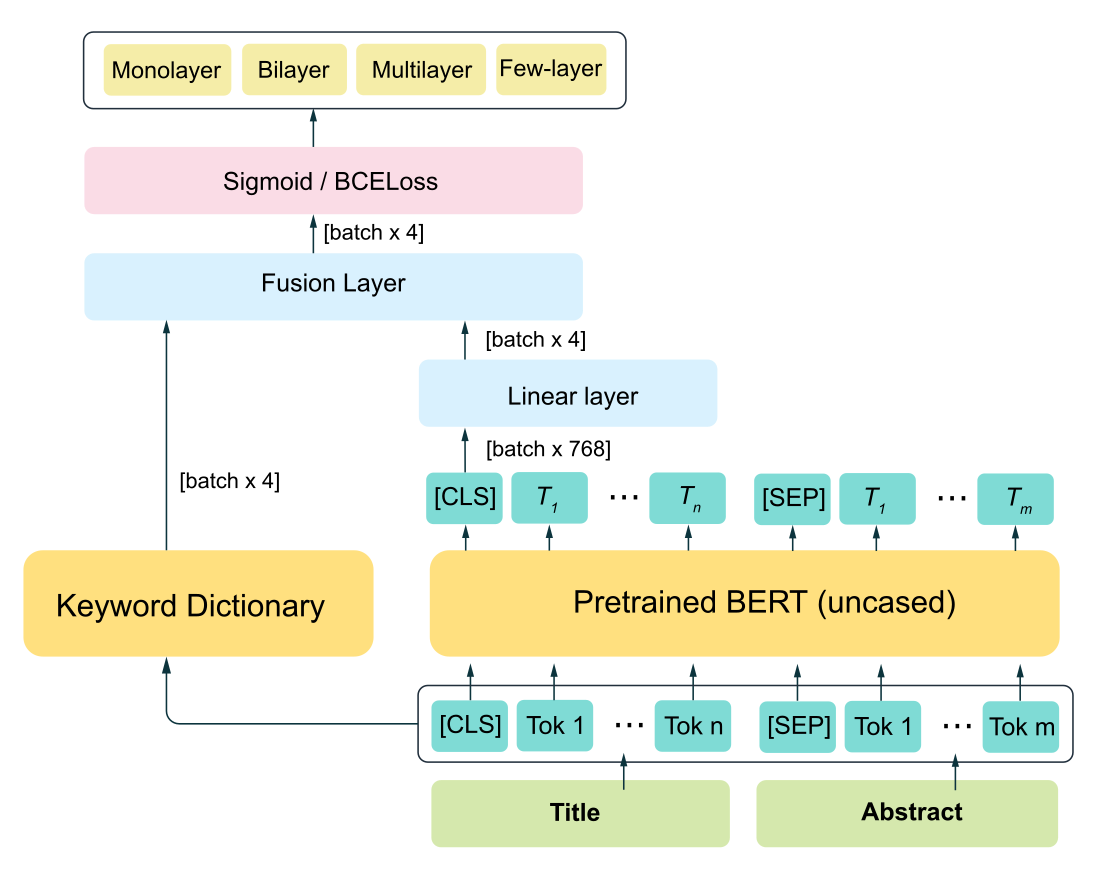


**Figure S1**. Architecture of abstract classification. A title and an abstract are combined as input for the pre-trained BERT model. The linear layer atop the pre-trained model reduces the dimension of the [CLS] token from 768 to 4 and then integrates it with the keyword dictionary in the fusion layer. Following the fusion layer, we employ a sigmoid function for prediction and BCELoss for training. The predicted multilabels include monolayer, bilayer, multilayer, and few-layer.


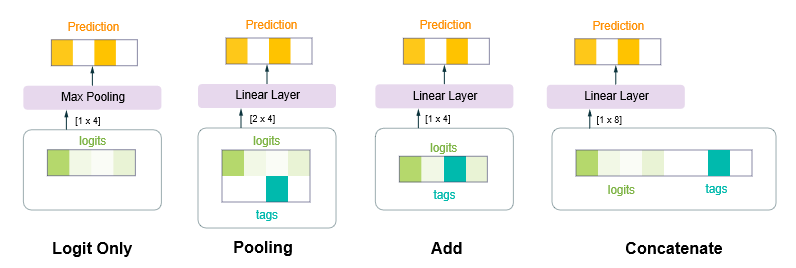


**Figure S2**. Types of Fusion layer in the abstract classification. The types of the fusion layer include logit only, pooling layer, add, and concatenate.


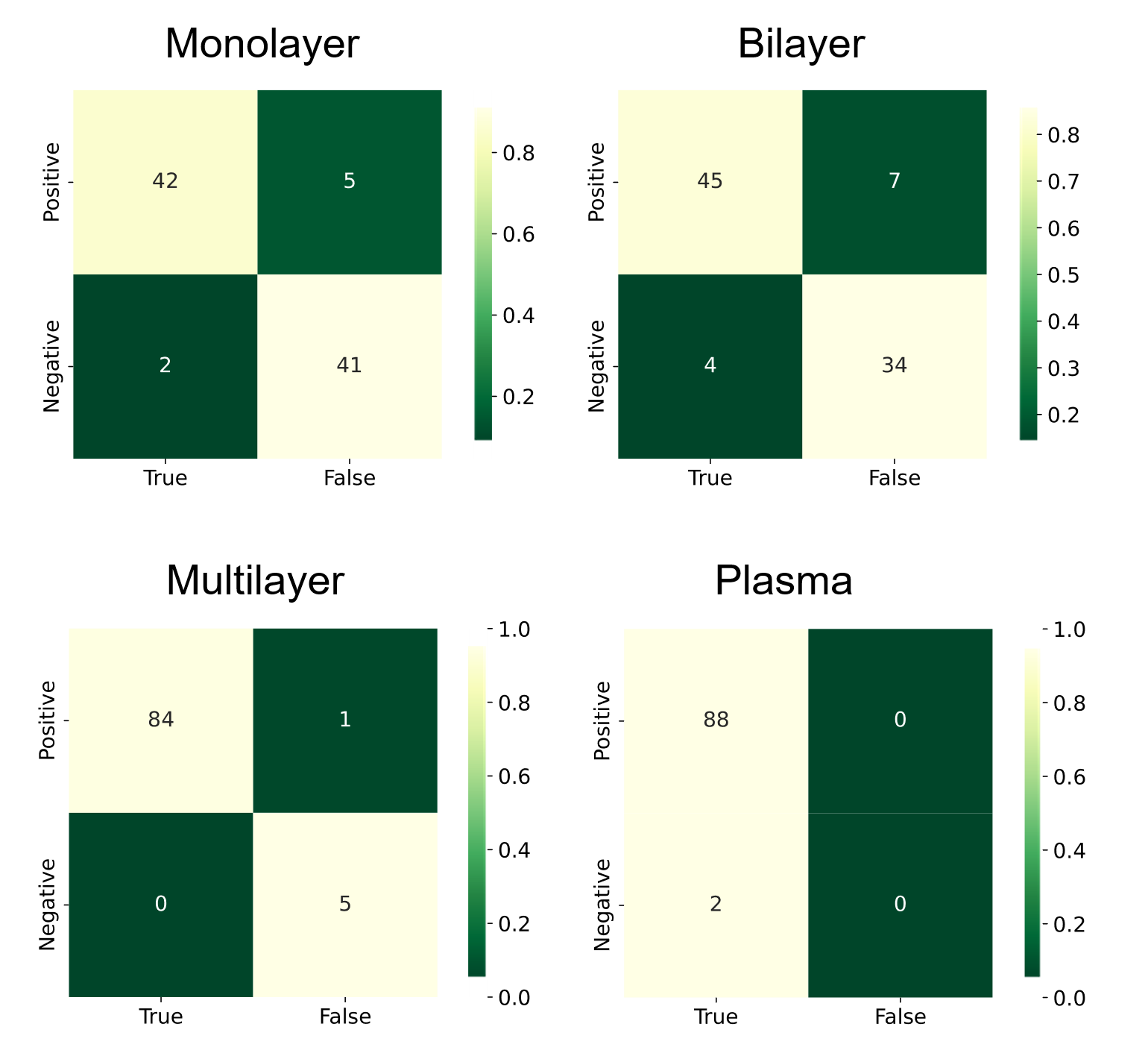


**Figure S3**. Confusion matrix of abstract classification.

- 1. Named Entity Recognition (NER)
     1. Data preprocessing

Data were extracted from the methods sections of papers focusing on materials such as MoSe_2_, WS_2_, WSe_2_, MoS_2_, hBN, graphene, and carbon nanotubes. Using regular expressions, web-scraped articles lacking methods sections were removed, and in-text and reference section citations were removed. Subsequently, each methods section was divided into individual sentences and tokenized, with each token corresponding to a label acquired from manual annotation: 0: N/A, 1: Substrate, 2: Catalyst, 3: Temperature, 4: Pressure, 5: Time, 6: Argon, 7: Hydrogen, 8: Precursor Material, 9: Precursor Temperature, 10: Precursor Flow, 11: Precursor Weight. We curated a manual annotation NER dataset prepared by our domain experts using LabelStudio. Annotators labeled tokens with one of the eleven categories from 1: Substrate to 11: Precursor Weight. Tokens that were left unlabeled were assigned the default label 0: N/A. To verify inter-annotator consistency in labeling, we computed the Fleiss’ kappa coefficient on four passages that were each annotated by two distinct annotators. The computed Fleiss’ kappa coefficient was 0.74, suggesting a high inter-annotator consistency.

- - 1. Unit standardization and normalization

Our preprocessing workflow incorporates a systematic unit conversion module that standardizes the diverse unit representations commonly found in materials science literature. Through rule-based text replacement operations, the system converts varied expressions of identical units (e.g., "ml·min⁻¹", "standard cubic centimeters per minute" → "sccm") into consistent forms, while simultaneously expanding chemical symbols and standardizing process terminology. This normalization step reduces vocabulary sparsity and ensures semantic consistency across the corpus, thereby facilitating more accurate named entity recognition and parameter extraction from scientific texts.

While our current regex-based approach for numerical value normalization proves effective for common formats, it can be fragile when confronted with unconventional unit spellings or complex textual descriptions of ranges and uncertainties. Future work would benefit significantly from integrating dedicated scientific unit parsing libraries, such as Pint or quantulum3, which would create a more robust and flexible normalization module capable of handling the full diversity of quantitative expressions encountered in scientific literature, further improving both the quality and completeness of the extracted database.

- - 1. The Model Architecture

The NER model employs a two-stage training approach to ensure robust performance in the specialized domain of materials science. Initially, we leverage the BertForTokenClassification architecture, which is built upon a BERT model that has been pre-trained on large general-domain text corpora including Wikipedia and BookCorpus. This foundational pre-training enables the model to capture essential language patterns, grammatical structures, and contextual relationships that are fundamental to natural language understanding.

The processed data was subsequently fed through this BERT layer, utilizing the BertForTokenClassification model which combines the pre-trained BERT model with an added token classification layer. Following the general pre-training, we perform domain-specific fine-tuning using our manually annotated dataset, which was specifically curated from the methods sections of journal articles focusing on 2D materials and carbon nanotubes. This fine-tuning step is crucial for adapting the model to the specialized terminology, abbreviations, and contextual nuances unique to materials synthesis research. The fine-tuning process allows the model to learn domain-specific entity recognition patterns while retaining the broad linguistic competence acquired during pre-training. CrossEntropyLoss was employed for processing the loss during both the fine-tuning and training phases. A schematic representation of the model architecture can be found in Figure S4.

While our approach focuses on BERT-based token classification for entity extraction, alternative strategies for structured synthesis knowledge extraction have also been explored. To avoid challenges regarding computational expense and the lack of LLMs’ mechanistic knowledge, recently Dagdelen et al. (2024) demonstrated the use of LLMs for structured materials entity relation extraction through several rounds of finetuning to output hierarchical JSON-like relationship structures^[6]^ . This complementary line of research highlights broader directions for extracting and organizing scientific knowledge, while our approach emphasizes lightweight models with transparent intermediate outputs suited to the specialized context of CVD synthesis literature.

- - 1. Hyperparameter Settings

The optimizer employed was the AdamW optimizer, where “W” at the end indicates it included weight decay on selected parameters. Additionally, the model incorporated a learning rate scheduler. The two options examined for the scheduler were the cosine and cosine with hard restarts learning rate schedulers. Hyperparameter tuning was conducted using Bayesian Optimization on the model, adjusting the number of epochs, learning rate, and the maximum threshold for gradient clipping.

- - 1. Evaluation Metrics

We measured the performance of the predictive model using the macro-averaging F1 score metric. The results are shown as confusion matrices in Figure S5.

- - 1. Challenges in Quality Assessment and Growth Optimization of 2D Materials

Extracting optimized synthesis parameters for 2D materials from literature is crucial for advancing materials development and enabling higher-performance device applications. Although computational models and data-driven approaches offer significant promise for this optimization, their effectiveness relies heavily on the quality and objectivity of the underlying data. However, inconsistencies and subjectivity in how synthesis outcomes are reported in the literature present a significant methodological challenge to reliably identifying optimal growth conditions.

Although our present work systematically extracts and organizes synthesis parameters from published articles, a major limitation arises from the fact that both text and figure data (e.g., Raman spectra or SEM images) are inherently restricted to what individual authors choose to report, and therefore cannot fully capture optimized growth conditions. This is a broader limitation of the available literature rather than of text mining techniques themselves. As a result, comparisons across studies may remain incomplete, potentially overlooking optimal synthesis strategies that are not fully documented. Looking ahead, we anticipate that community-driven databases linking synthesis parameters with standardized quality metrics will emerge in the field, and our framework provides the structural foundation to integrate such resources once they become available.

By compiling objective quality metrics derived from actual experimental data, it becomes possible to more equitably evaluate and compare diverse synthesis recipes reported across the literature. This curated and structured dataset can then serve as a robust foundation for training optimization models—such as Bayesian optimization or gradient boosting trees—to extract prior knowledge and ultimately recommend synthesis parameters that are more likely to yield 2D materials of superior crystallinity.


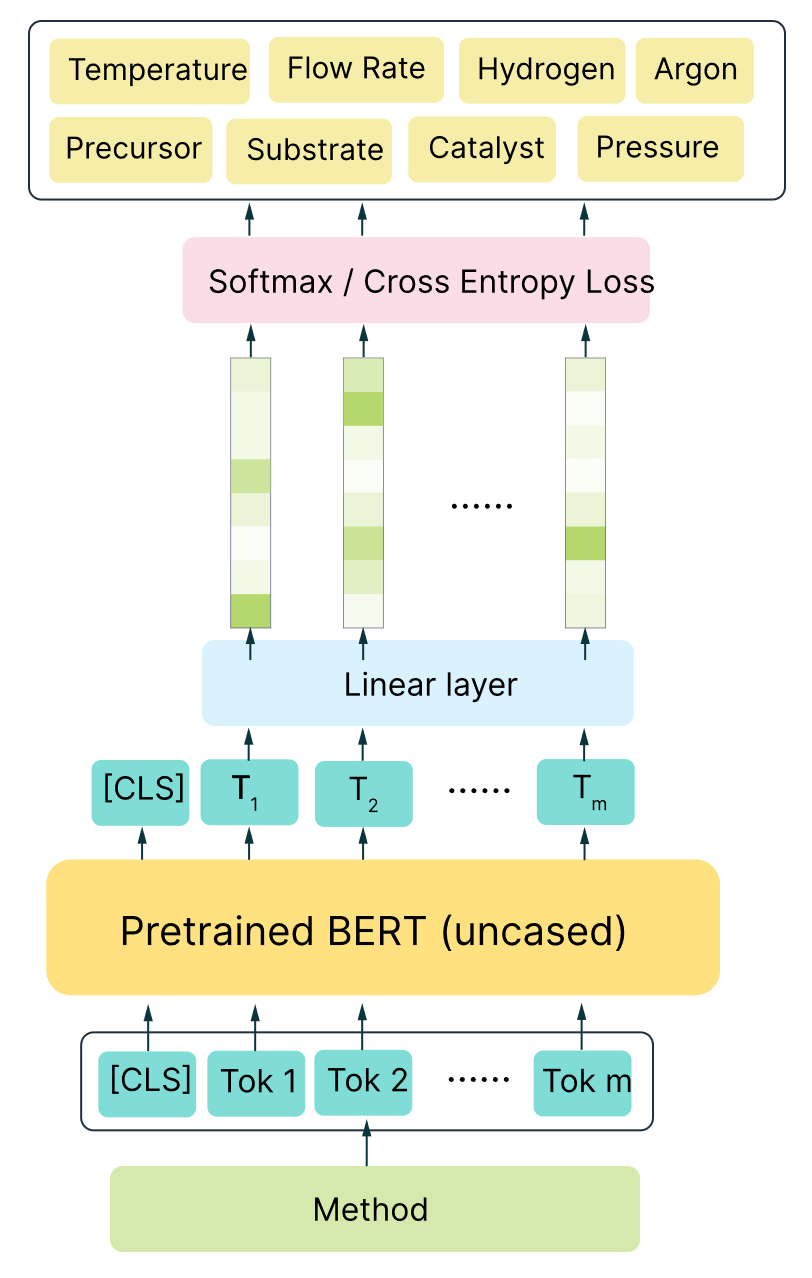


**Figure S4**. Architecture of named entity recognition (NER) model.


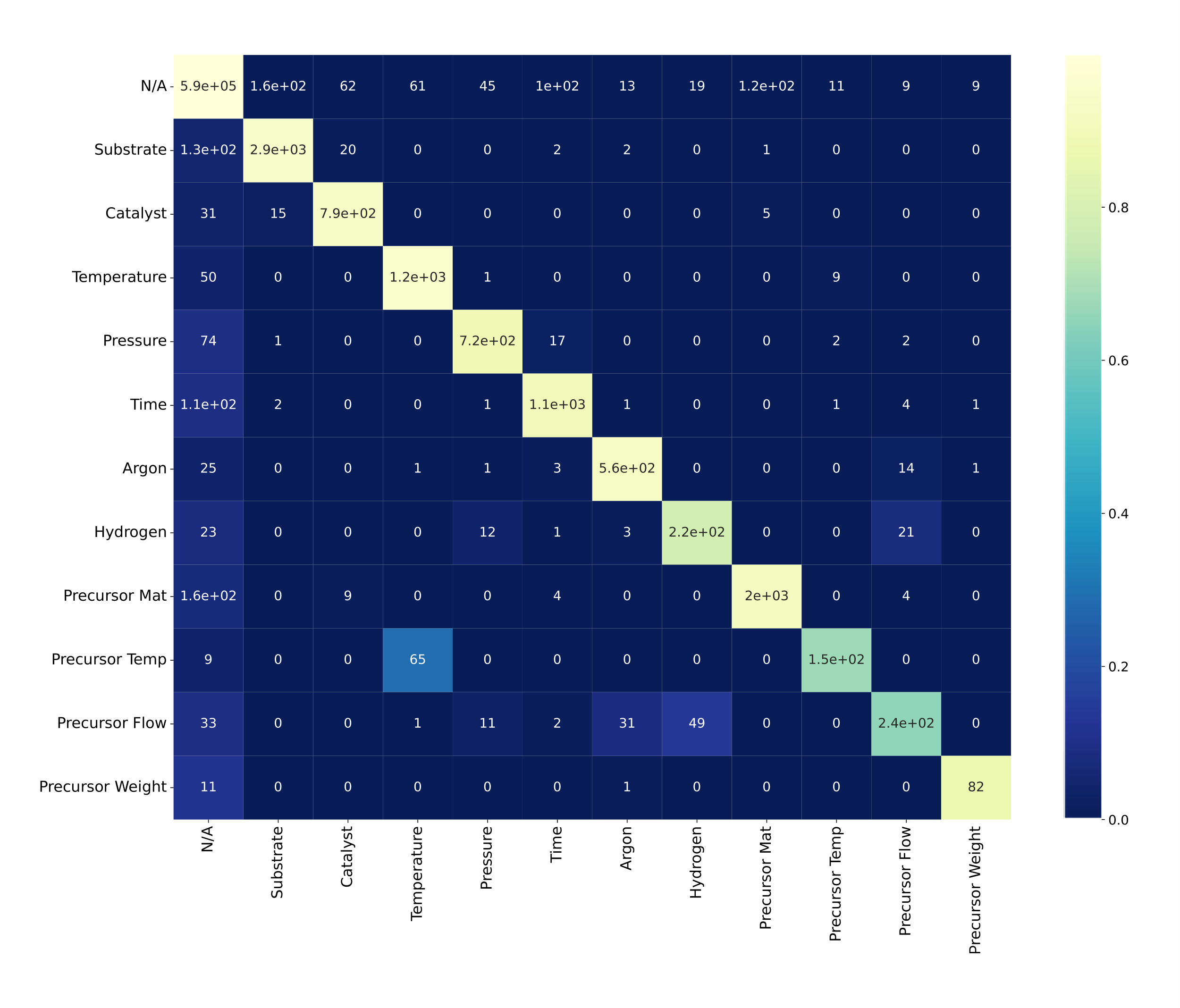


**Figure S5**. Confusion matrix for trained named entity recognition (NER).


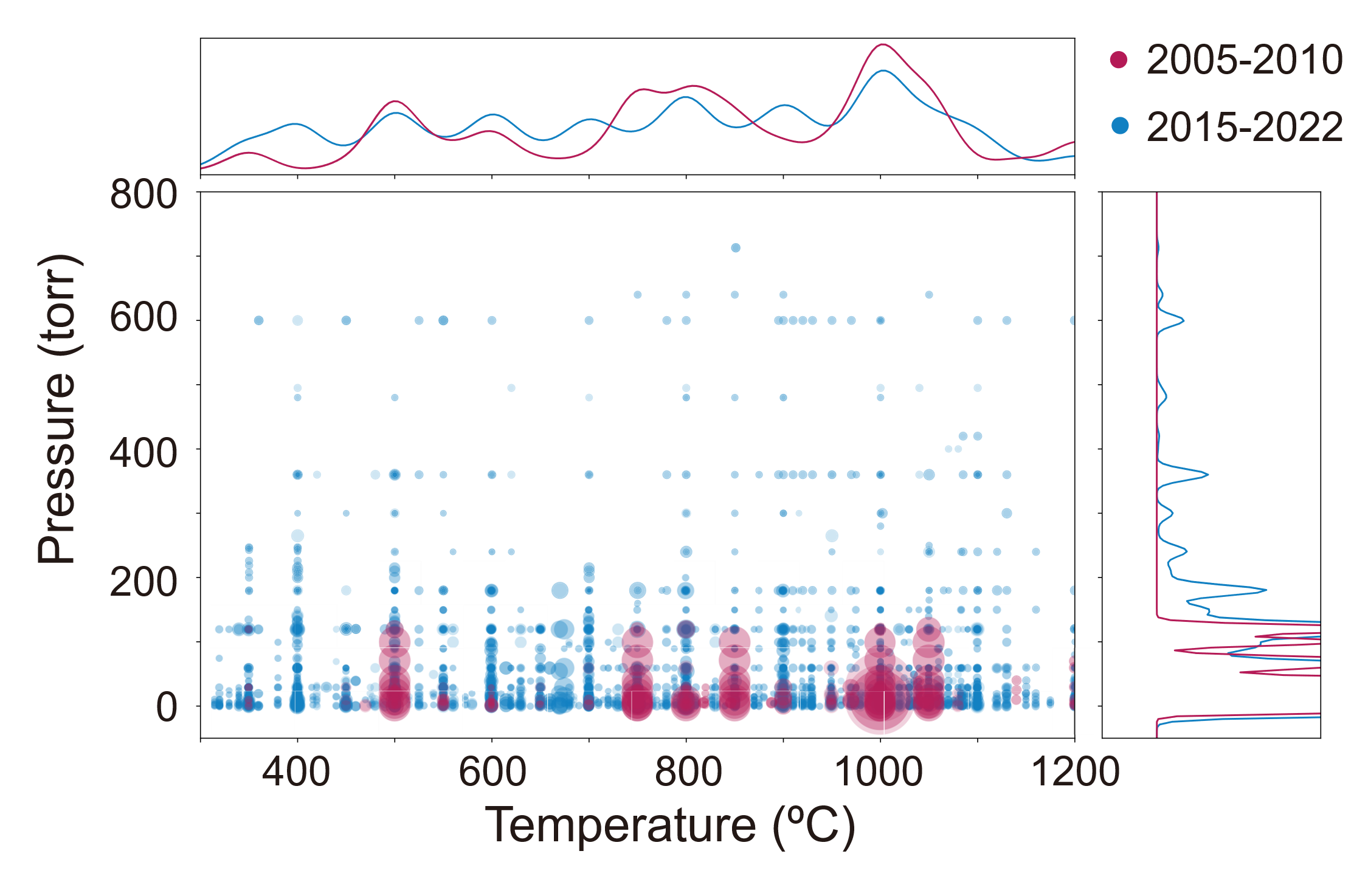


**Figure S6.** Scatter plot of temperature versus pressure for the data extracted by the NER model.

- 1. Extractive Question Answering (EQA)
     1. The Model Architecture

The architecture of the EQA models, as shown in Figure S7, is based on the BERT model framework and employs a multi-stage training approach to ensure robust performance in materials science question answering. The EQA models operate by adding two distinct neural network head layers to the BERT base, predicting the most probable start and end tokens for an answer. We utilized BERT and SciBERT models pre-trained on the SQuAD1.1 dataset and a combination of SQuAD2.0 and QuAC datasets, respectively, which are widely used benchmarks for reading comprehension and question answering. Additionally, we adapted MatSciBERT to perform EQA by starting with MatSciBERT weights (a BERT-based model pre-trained on materials science literature) and randomly initialized question answering heads, then fine-tuning parameters by training on the SQuAD1.1 dataset to further enhance domain-specific performance.

Related work has demonstrated that domain-specific pretraining can significantly improve QA-driven entity extraction. For instance, Huang and Cole (2022) pretrained BatteryBERT models^[22]^ and applied them to battery entity extraction with extractive question answering. Building on this paradigm, we extend the approach to 2D materials by applying BERT, SciBERT, and MatSciBERT for entity extraction, and further leverage our framework to generate insights by analyzing the historical distribution of 2D synthesis recipe parameters.

SQuAD (Stanford Question Answering Dataset) is a reading comprehension dataset from volunteers posing questions using Wikipedia as context, and QuAC (Question Answering in Context) is a student-teacher dialog dataset also using Wikipedia as context. BertForQuestionAnswering is a BERT model fine-tuned for question answering. SciBERT is a BERT variant trained on scientific literature, and MatSciBERT is a BERT variant trained on materials science literature. Our approach leverages these state-of-the-art pre-trained language models to maximize extraction accuracy for synthesis parameters and answers to technical questions.

- - 1. Fine-Tuning and Evaluation of EQA Models

We fine-tuned the MatSciBERT EQA model based on the SQuAD1.1 dataset. For comparison purposes, we manually annotated 41 journal articles using the question list in Table S1. We performed predictions on the manually annotated articles using the BERT, SciBERT, and MatSciBERT QA models, comparing their predictions to manual annotations by domain experts. For each of the three models, we selected an answer confidence threshold below which we output empty predictions by optimizing the F1 score on test data through a grid search (optimal thresholds were BERT: 0.0002, SciBERT: 0.006, MatSciBERT: 0.1). During the fine-tuning process of the MatSciBERT model, we employed a linear scheduler without warmup, a batch size of 8, and an AdamW optimizer with a learning rate of 2E-5. By evaluating the F1 score on our manually annotated dataset after each epoch, we determined that optimal performance was achieved after 2 epochs, with an overall F1 score of 0.287, followed by a decline in performance thereafter. Consequently, we utilized a model trained on 2 epochs for EQA prediction. The model is available at: <https://huggingface.co/rachen/matscibert-finetuned-squad-pytorch>.

- - 1. Evaluation of F1 Score for Question Answering Responses

We evaluate the F1 score for question answering according to the original SQuAD1.0 paper, where we count the number of overlapping words between the prediction and gold answer. Therefore, the F1 score is computed as

$F1=2\cdot\frac{precision\cdot recall}{precision+recall}$,

Where

$$precision=\frac{\# common words}{\# words in prediction}, recall=\frac{\# common words}{\# words in truth}.$$

- - 1. Prospective Materials Science Insights from Large-Scale Pattern Analysis

Beyond retrospective data extraction and visualization, our framework demonstrates prospective scientific value by identifying unexplored parameter spaces through macro-scale pattern analysis. The systematic extraction of synthesis parameters across thousands of experiments enables us to recognize emergent trends and optimization opportunities that would be difficult to discern from individual publications.

The intermediate pressure gap observed in Figure 3b exemplifies this capability. The bimodal distribution clustering at ~760 Torr (APCVD) and ~1 Torr (LPCVD), with conspicuous underpopulation in the 50–200 Torr regime for TMDs, suggests an unoptimized parameter space that may combine APCVD-like precursor availability with LPCVD-like uniformity while avoiding turbulence and vacancy formation. This hypothesis is grounded in fundamental CVD physics: atmospheric pressure can introduce turbulent fluid dynamics and complex boundary layer effects, while ultra-low pressure reduces chalcogen partial pressure, potentially leading to vacancy defect formation in TMDs.

Recent experimental validation supports this interpretation. Abidi et al. (2024)^[19]^ demonstrated that 1 Torr LPCVD produces sulfur vacancy-rich MoS₂ with photoluminescence intensity 300× lower than atmospheric pressure samples, attributing this to "sulfur vacancy induced defect rich crystals primarily attributed to the kinetics of the growth conditions." Critically, Moses et al. (2025)^[21]^ achieved uniform epitaxial monolayer MoS₂ films with minimal bilayer nucleation across 2-inch wafers using 50 Torr MOCVD—falling precisely within the identified gap—while maintaining high optical quality. Additionally, Paidi et al. (2024)^[20]^ confirmed that APCVD growth operates in a diffusion-limited regime with characteristic triangular morphology, contrasting with LPCVD's reaction-limited, polycrystalline growth. The scarcity of 50–200 Torr studies likely reflects practical equipment barriers (throttle valve requirements for pressure control) rather than fundamental limitations, suggesting this regime warrants systematic exploration for future TMD synthesis optimization.


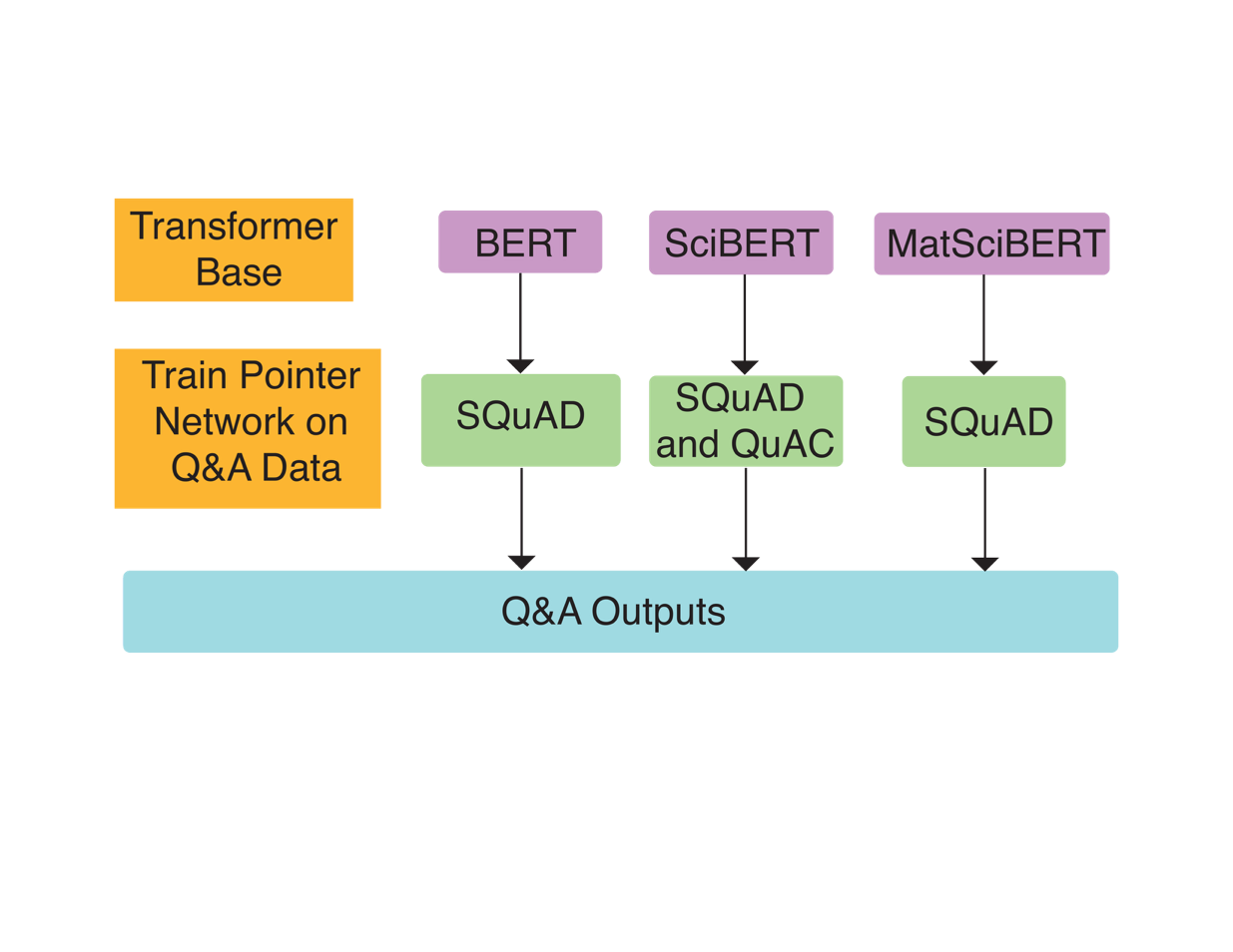


**Figure S7.** Extractive Question Answering (EQA) model architecture.

**Table S1.** Questions used in extractive question answering.

| **Named Entity** | **Question Used** |
| --- | --- |
| CVD | What is CVD? |
| Layer | How many layers were there? |
| Substrate | What type of substrate was used? |
| Precursor | What types of precursors were used? |
| Temperature | What was the temperature of the substrates? |
| Time | How long was the growth time in the heating zone? |
| Pressure | What was the chamber pressure? |
| Characterization | What type of characterization was used? |
| Flow Rate | What were the gas types and flow rates? |

**Table S2.** Average length of gold, BERT, SciBERT, and MatSciBERT answers for nonzero question responses, calculated separately for each named entity.

| **Named Entity** | **Average Length of Nonzero Gold Answer** | **Average Length of Nonzero BERT Answer** | **Average Length of Nonzero SciBERT Answer** | **Average Length of Nonzero MatSciBERT Answer** |
| --- | --- | --- | --- | --- |
| CVD | 27.7 | 27.1 | 49.8 | 25.1 |
| Layer | 20.4 | 8.8 | 50.2 | 13.8 |
| Substrate | 17.5 | 11.0 | 41.2 | 10.2 |
| Precursor | 15.3 | 26.0 | 45.8 | 17.4 |
| Temperature | 15.7 | 10.8 | 47.1 | 10.6 |
| Time | 9.1 | 8.7 | 52.4 | 7.2 |
| Pressure | 13.3 | 12.5 | 32.8 | 11.1 |
| Characterization | 49.8 | 23.0 | 70.6 | 22.2 |
| Flow Rate | 37.1 | 23.4 | 38.0 | 22.3 |


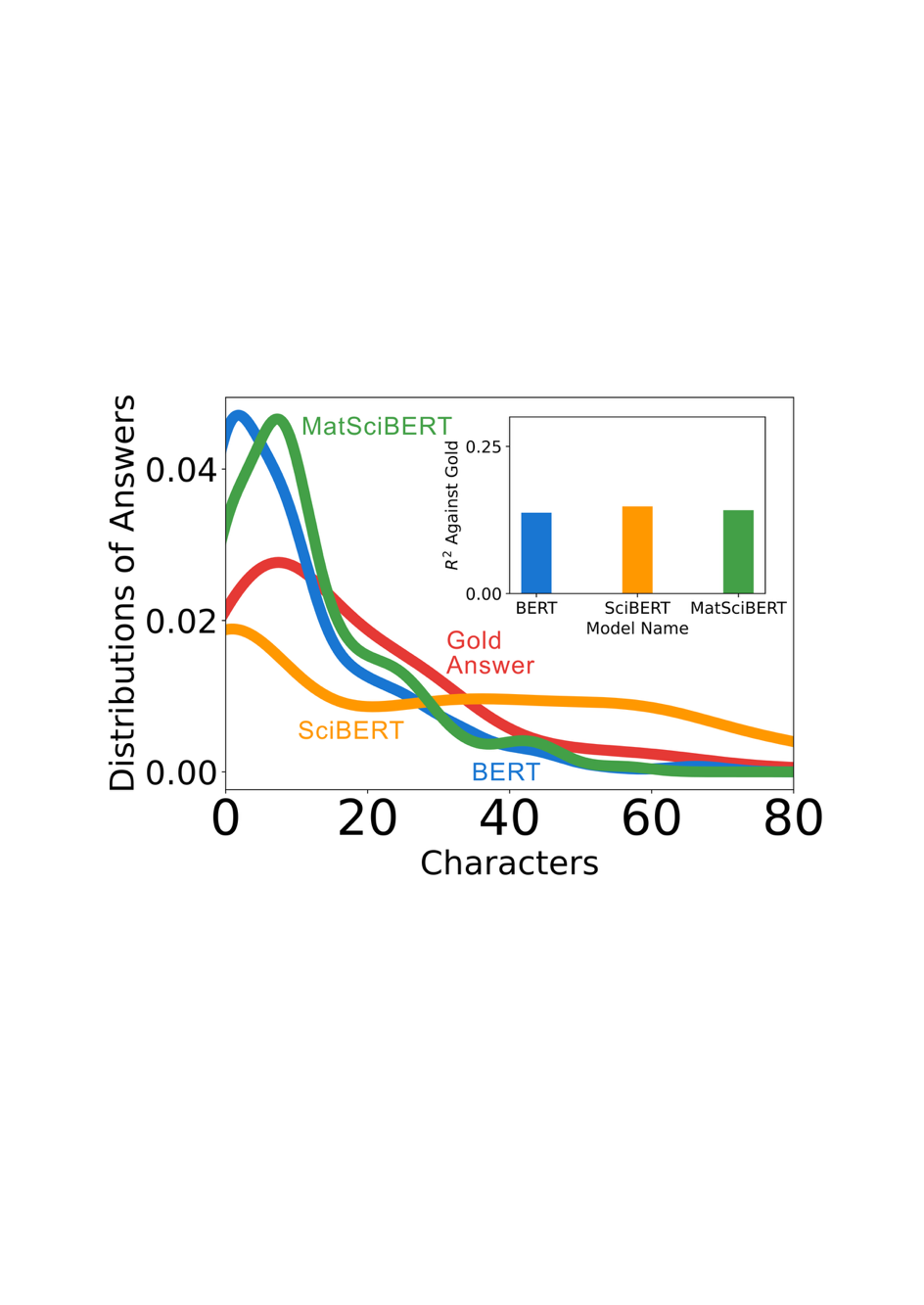


**Figure S8.** Kernel density estimation (KDE) plots representing the distributions of answer lengths for manually annotated, BERT, SciBERT, and MatSciBERT answers. The inset bar chart shows the correlation coefficient of the best fit line for answer lengths of BERT-based models against manually annotated answers.


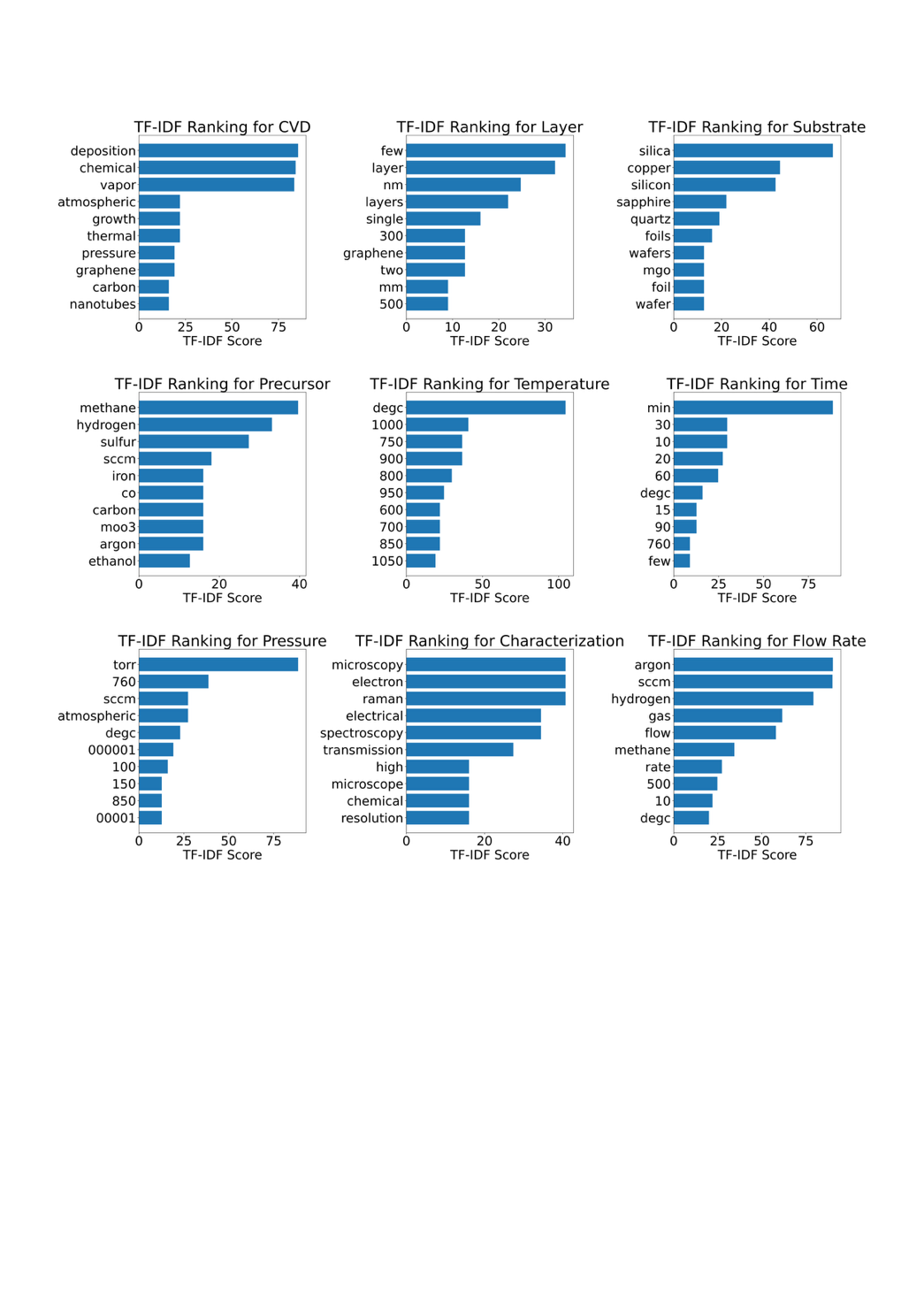


**Figure S9.** A TF-IDF analysis on the EQA outputs for each category. Rankings are calculated by performing a TF-IDF vectorization on all EQA outputs from one question and summing up the output vectors. Higher-ranked terms indicate words that are most uniquely representative within all responses of a question category, helping to highlight key concepts and parameters extracted by the model.


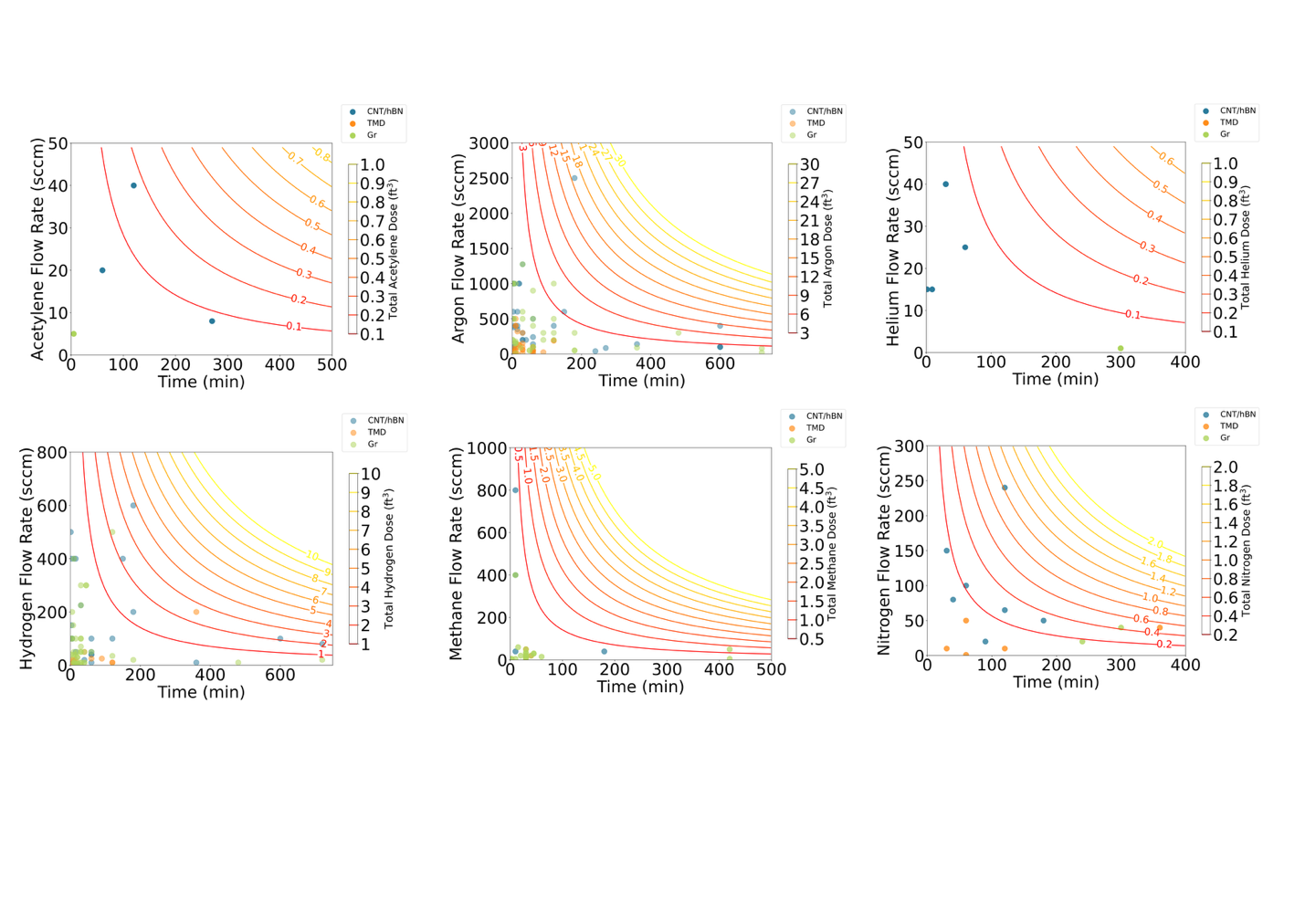


**Figure S10.** Scatterplot of experiment duration versus gas flow rate for six common precursor or carrier gases (acetylene, argon, helium, hydrogen, methane, and nitrogen). Contour lines indicating total dose of gas, calculated as growth time times flow rate, are overlaid on the scatter plots. Experiments with a low and high range for durations are plotted twice on each corresponding gas plot. Experiments with a duration of 24 hours (1440 minutes) or more are excluded as errors.


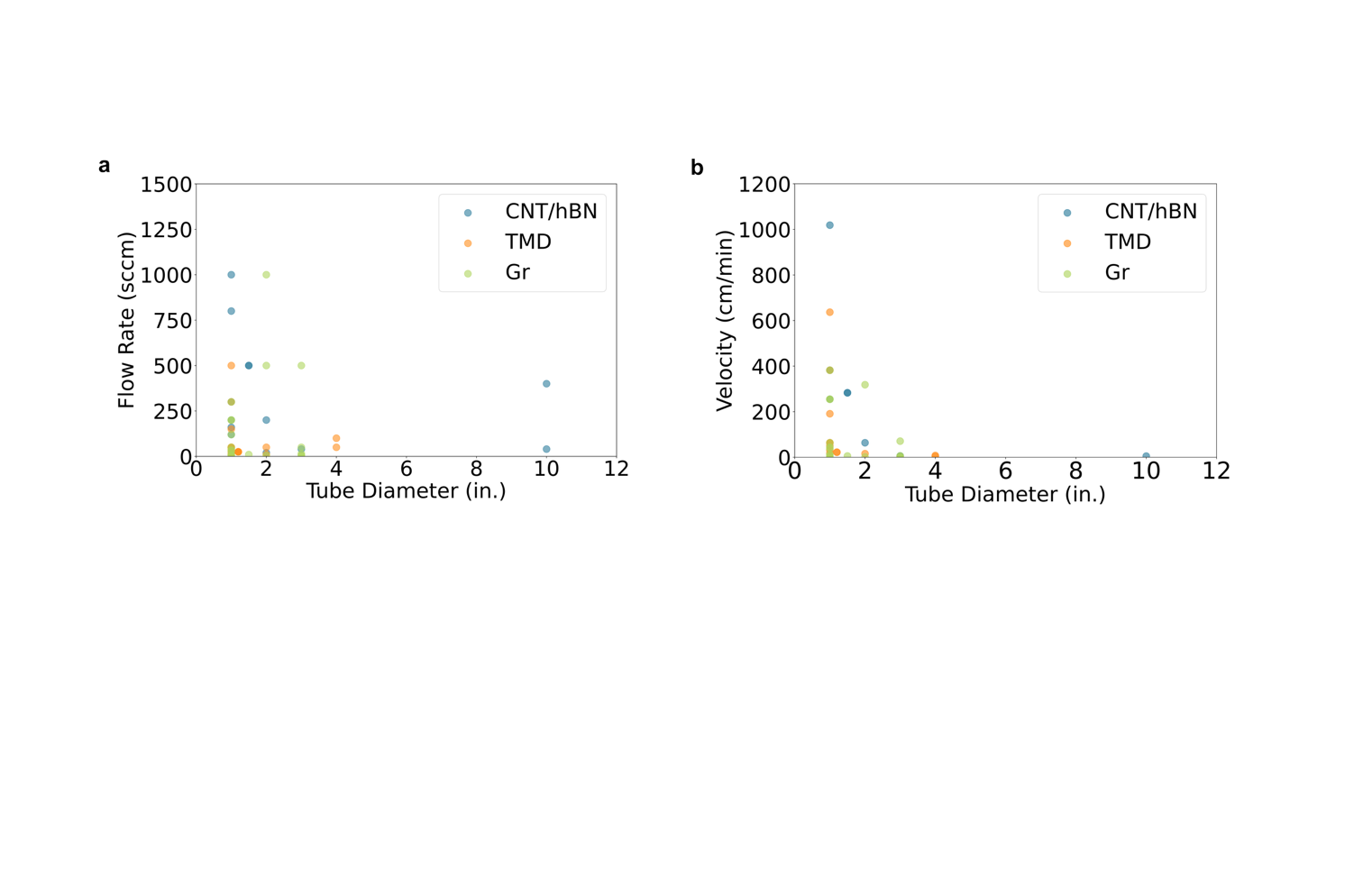


**Figure S11.** (a) Scatterplot of quartz tube diameter (queried using “What was the diameter of the quartz tube?” with the MatSciBERT EQA model) and maximum flow rate. (b) Scatterplot of quartz tube diameter and velocity, computed as maximum flow rate divided by cross sectional area. Scatter points are colored with the associated material category. In both plots, we choose to examine maximum flow rate and assume that entrainment causes gases at lower flow rates to mix with gases of higher flow rates. We exclude predictions of quartz tube diameters exceeding 12 inches and assume they are errors.

- 1. Generative Models
     1. Multi-Document Summarization and LDA

In the context of multi-document summarization, we directly employed the PRIMERA model for generating summaries corresponding to grouped experimental sections from the literature on a specific material. We iteratively produced 100 summaries for each material category and performed Latent Dirichlet Allocation (LDA) using Gensim, an open-source Python library. This enabled us to cluster the topics present in the summaries through unsupervised learning. Visualization of LDA results displays an inter-topic distance plot based on principal component analysis, as well as further subdivision into subtopics, as shown in Figure S12. Using an unsupervised learning methodology, this analysis offers a preliminary categorization of the topics encompassed within the summaries and experimental sections of the literature. It should be noted that the accuracy of the results may be enhanced by augmenting the volume of textual data from the literature or refining the text through the implementation of specific methods or examples.


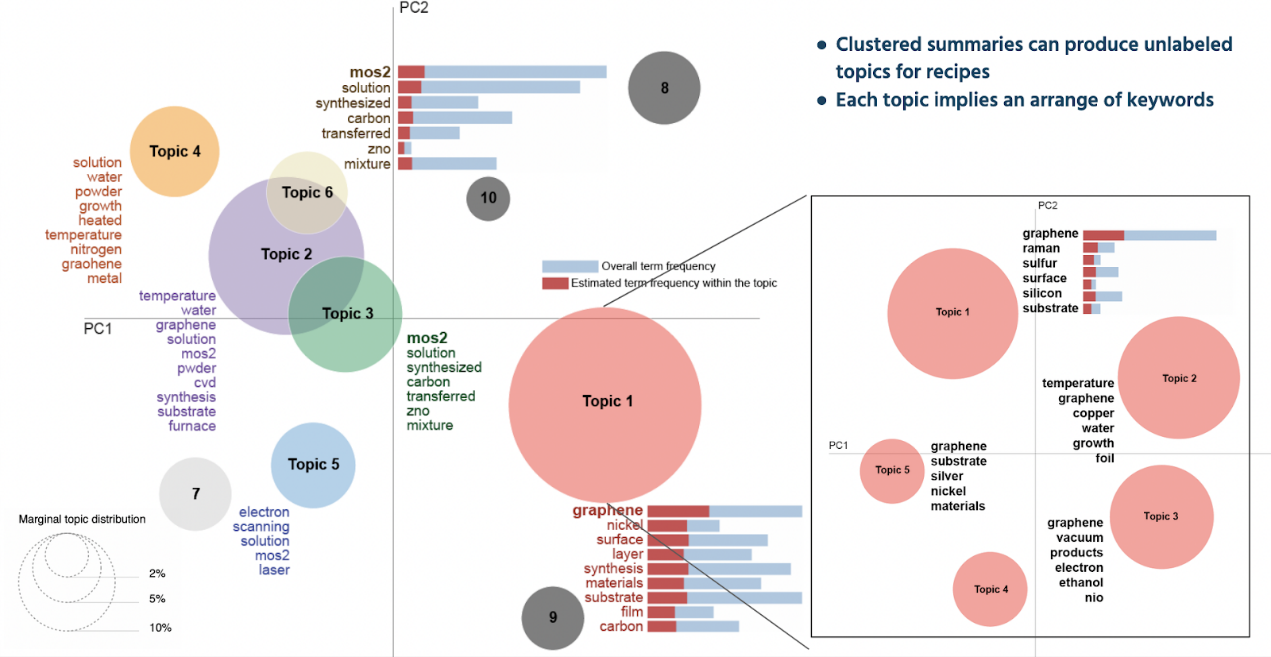


**Figure S12.** Topic clustering analysis of 2D material literature using the LDA model.

- - 1. Fine-Tuning Process and Hyper-parameter Settings

We fine-tuned the GPT-Neo model using the cleaned experimental sections from our dataset, assigning each sentence a material-year group label to achieve a structured output. Optimal hyperparameters for the GPT-Neo model, were a learning rate of 2E-5 and 50 epochs (for models with more than 100 epochs, training was time-consuming and possibly overfitting).

**Table S3.** Example of assigning material-year group labels to training text input.

| **Literature** | **Training input** |
| --- | --- |
| CNT Growth and Characterization  CNTs were grown in a custom-built hot-wall tube furnace, with a rapid sample insertion mechanism. First, the substrate was annealed to induce catalyst film dewetting and nanoparticle formation in a reducing atmosphere of hydrogen and helium (400 sccm H2/100 sccm He) at 775 ˚C (10 min ramp time and 10 min temperature hold). After the annealing step, the substrate was retracted from the reactor and held in an adjacent cold chamber while introducing the feedstock gas, ethylene (C2H4­), changing the gas mixture to the growth atmosphere (100 sccm C2H4/400 sccm He/100 sccm H2) at the same temperature. After 7 min, during which the gases and the humidity inside the tube furnace stabilize, the substrate was returned to inside the reactor. CNTs were characterized by scanning electron microscopy (SEM), using a Philips XL30FEG. | <\|startoftext\|>[CNT-3] CNT Growth and Characterization CNTs were grown in a custom-built hot-wall tube furnace, with a rapid sample insertion mechanism.[CNT-3] First, the substrate was annealed to induce catalyst film dewetting and nanoparticle formation in a reducing atmosphere of hydrogen and helium 400 sccm H2/100 sccm He at 775 degC 10 min ramp time and 10 min temperature hold. [CNT-3] After the annealing step, … [CNT-3] CNTs were characterized by scanning electron microscopy SEM , using a Philips XL30FEG.<\|endoftext\|> |

- - 1. Prompting Designs

We designed prompts following a uniform structure: "To synthesize [material], the [component], was", wherein the [material] placeholder was paired with one of the seven materials and the [component] placeholder was paired with one of the elements from {catalyst, substrate, temperature, pressure, characterization, application}. For each prompt input, we generated the output iteratively 100 times and recorded the results.


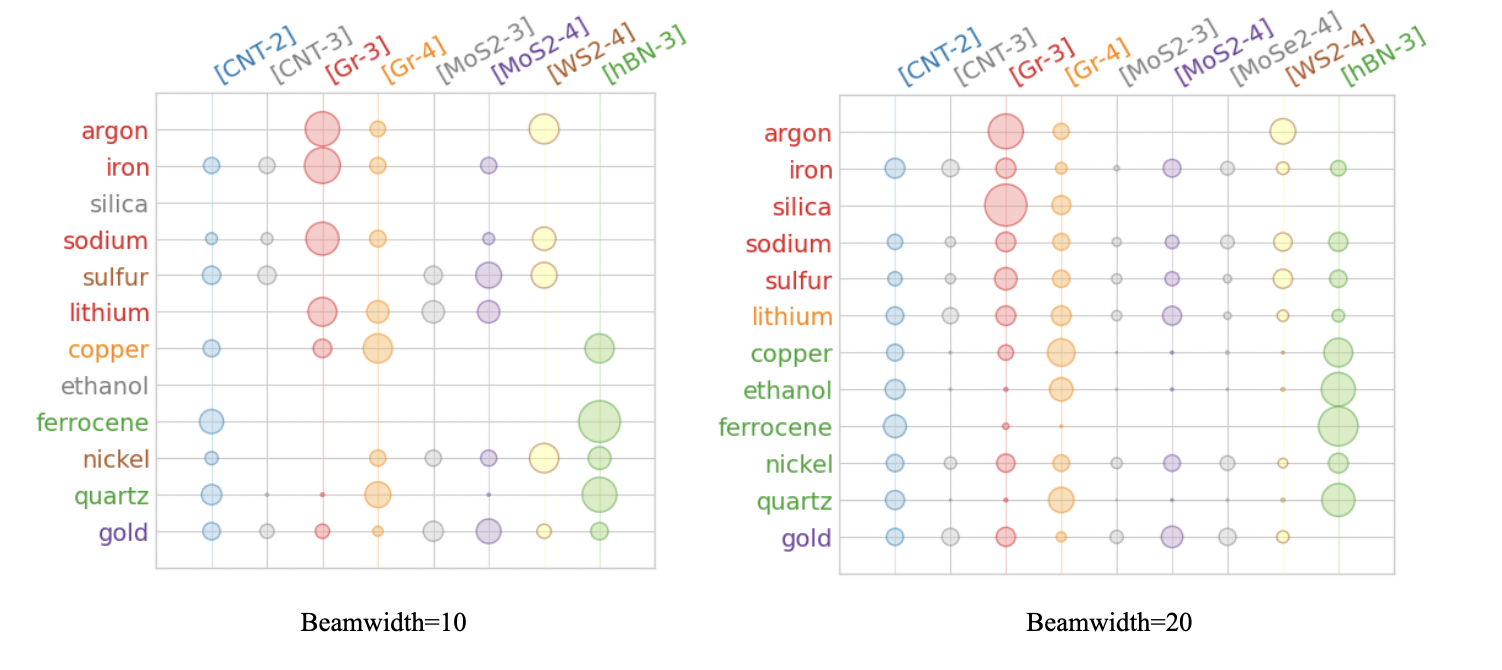


**Figure S13**. Comparison of termite plots with varying beamwidths using the GPT-Neo model. Beamwidth refers to the number of possible next-token candidates considered at each step during text generation. A larger beamwidth allows for more diverse outputs by exploring multiple possible continuations, while a smaller beamwidth results in more deterministic predictions. This comparison highlights how different beamwidth settings influence keyword distributions in the generated text.

Based on the analysis results shown in the above termite plots, the bubble size of each component-material pair indicates the probability of their co-occurrence in the same prompted output. Each word on the left side of the plot is colored based on the most related, i.e. most frequently co-occurrent with, material-year group. For example, keywords colored red (argon, iron, etc.) are most probable to show up together with [Gr-3] (Graphene literature data from 2011 to 2015) while gold is the only keyword colored in purple which links to [MoS_2_-4] (MoS_2_ data from 2016 to 2022). When increasing the ‘beamwidth’ parameter to generate prompted outputs, some hidden pairs can be revealed such as silica-[Gr-3] because a larger beamwidth supports better search for next generated words in longer sentences.

**Table S4.** Material group labels for termite plot analysis, showing Graphene, MoS₂, WS₂, MoSe₂, WSe₂, CNT, and hBN categorized into five time periods (before 2000, 2001–2005, 2006–2010, 2011–2015, and 2016–2022).

| Before 2000 | 2001-2005 | 2006-2010 | 2011-2015 | 2016-2022 |
| --- | --- | --- | --- | --- |
| Group Label 0 | 1 | 2 | 3 | 4 |

- - 1. Future Outlook: Potential Integration with Retrieval-Augmented Generation (RAG)

Our generative models excel at surfacing validated parameter patterns that inform knowledge transfer, positioning our framework as a decision-support tool for adapting proven synthesis strategies. Looking forward, a promising direction is integrating our database with a Retrieval-Augmented Generation (RAG) system, as conceptualized in Figure S14, to transform it into a dynamic engine for evidence-based hypothesis generation.


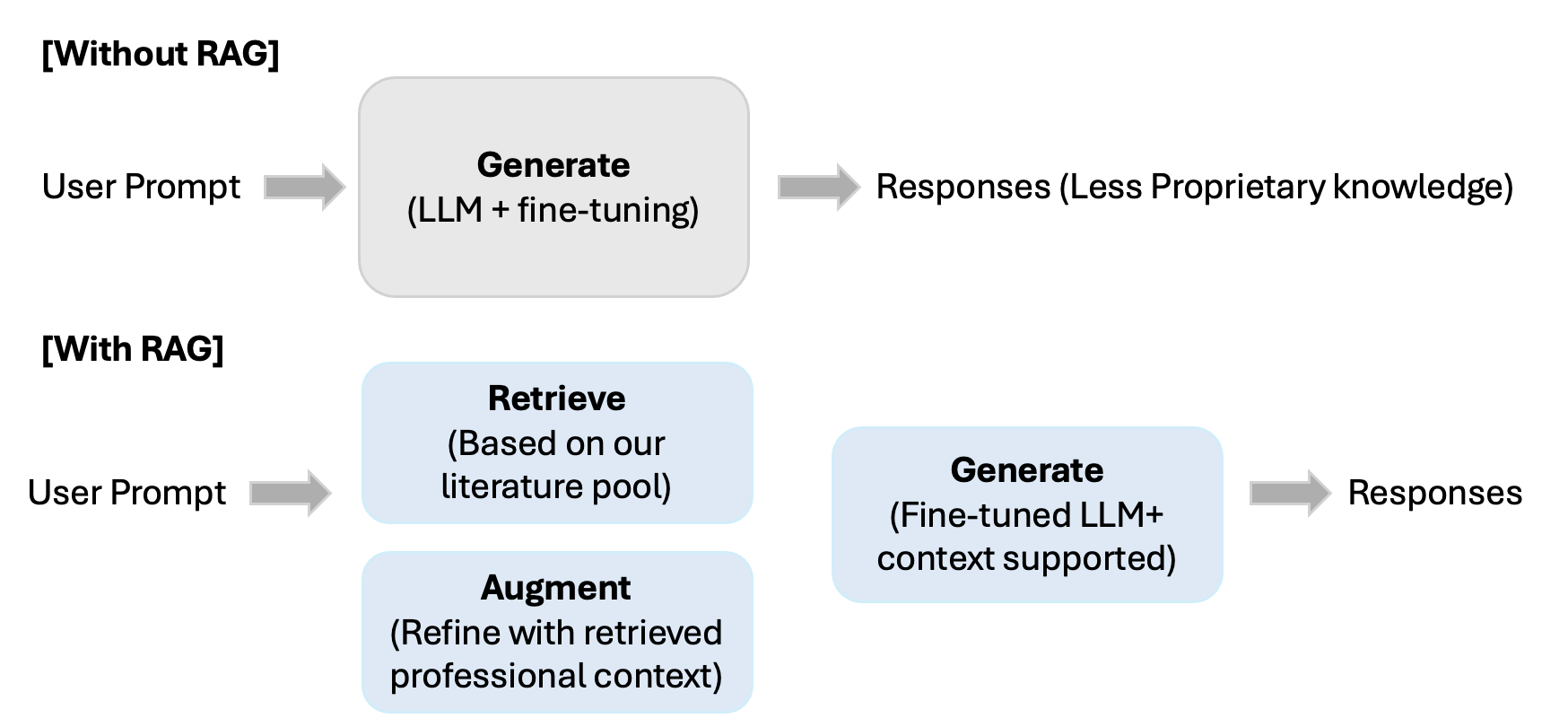


**Figure S14.** Proposed integration of Retrieval Augmented Generation (RAG) into the pipeline to enable researchers to query validated synthesis data and adapt proven parameter sets, supporting targeted, evidence-based knowledge transfer across material systems.

- - 1. Readability

The Kincaid readability test, formally known as the Flesch–Kincaid Grade Level, is a widely used metric for assessing how easy or difficult a passage of English text is to understand. It calculates a grade-level score based on sentence length and word complexity, aligning this score with U.S. educational grade levels—for example, a score of 7 indicates the text should be understandable to a typical seventh-grade student. The test was originally developed to help ensure that technical and educational materials were accessible to their intended audiences, and it remains useful for authors seeking to tailor scientific writing to broader or more specialized readers.


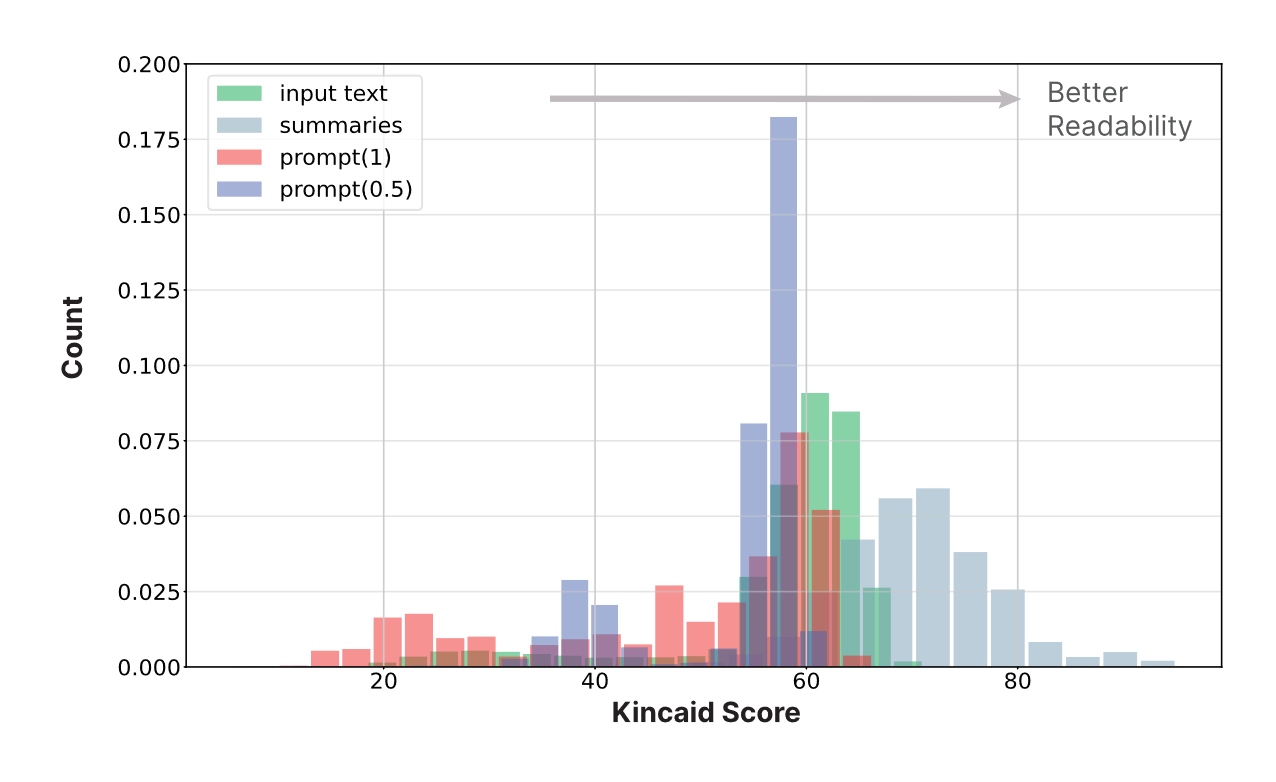


**Figure S15.** Readability based on the Kincaid score for input and output text for multi-document summary model and prompting, where ''(0.5)/(1)'' indicates randomness of generative setting parameters.

1. Discussion
   1. Validation Approach and Limitations

While our current study does not include direct wet-lab experimental validation, our methodology incorporates domain expertise as a form of semi-validation throughout the data extraction and analysis process. Our experienced annotators conducted careful screening of synthesis recipes and applied prior knowledge of 2D materials synthesis to filter plausible parameter combinations during both the NER and EQA stages. Additionally, extracting quantitative growth outcomes from literature presents significant challenges, as many studies report results qualitatively or with subjective assessments that make objective cross-study comparisons difficult. Nevertheless, we recognize that experimental validation represents an essential future direction for fully demonstrating the practical utility of our extracted parameter database and establishing its reliability as a resource for the materials synthesis community.

- 1. Substrate and Precursor Evolution in TMD Synthesis

Our analysis highlights clear chronological phases in CVD growth methodology. According to Figure S16 for MoS₂ synthesis evolution, studies before 2005 primarily utilized the direct reaction of molybdenum and sulfur elements on quartz substrates, marking the early exploratory stage of precursor chemistry. Between 2005 and 2010, there was a notable pivot toward the use of hydrogen sulfide (H₂S) on gold substrates, motivated by the need for improved crystallinity and lower synthesis temperatures. In the 2010–2015 period, silica (SiO₂) became the substrate of choice, coinciding with widespread adoption of molybdenum trioxide (MoO₃) and sulfur powder as precursors. This transition reflected crucial thermodynamic benefits: MoO₃ offers superior vapor pressure control and enables more uniform metal atom distribution than elemental molybdenum. Most recently, from 2015 to 2022, synthesis protocols have matured further, with research optimizing MoO₃/molybdenum–sulfur combinations across a variety of substrates including silica, quartz, and alumina. This progression reflects a growing body of experimental knowledge regarding substrate compatibility and thermal expansion considerations.

Additionally, WS₂ synthesis exhibited remarkably similar evolutionary patterns to MoS₂, confirming the transferability of synthetic knowledge across TMD families. However, MoSe₂ development began relatively late (2010-2015), reflecting the additional challenges associated with selenium handling and its lower vapor pressure compared to sulfur. Most intriguingly, WSe₂ synthesis showed distinct substrate preferences, with sapphire substrates being more prevalent than in other TMD systems. This preference stems from WSe₂'s requirement for higher growth temperatures (typically 750-850°C) compared to MoS₂ (650-750°C), WS₂ (600-800°C), and MoSe₂ (600-700°C). The thermal stability and lattice matching properties of sapphire make it particularly suitable for high-temperature WSe₂ synthesis. Additionally, our analysis reveals that WSe₂ synthesis uniquely employs tungsten hexacarbonyl [W(CO)₆] as an alternative metal precursor, providing greater process flexibility compared to oxide-based precursors. This diversity in precursor options reflects the more complex reaction pathways required for selenide formation compared to sulfide synthesis.


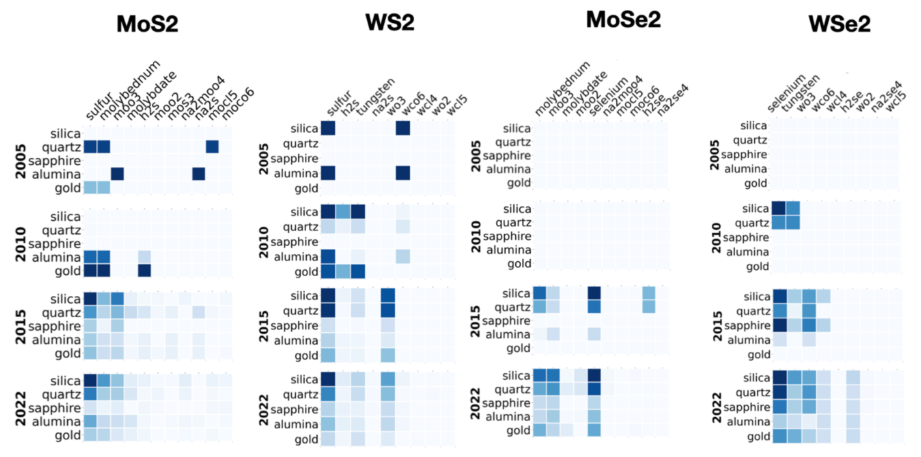


**Figure S16**. Heatmaps of substrate–precursor usage for MoS₂, WS₂, MoSe₂, and WSe₂ syntheses across four time periods, illustrating the chronological evolution of CVD growth strategies.

Reference

[1] L. Zhu, J. Tang, B. Li, T. Hou, Y. Zhu, J. Zhou, Z. Wang, X. Zhu, Z. Yao, X. Cui, K. Watanabe, T. Taniguchi, Y. Li, Z. V. Han, W. Zhou, Y. Huang, Z. Liu, J. C. Hone, Y. Hao, Artificial Neuron Networks Enabled Identification and Characterizations of 2D Materials and van Der Waals Heterostructures, *ACS Nano*, **2022**, *16*, 2721.

[2] O. Kononova, T. He, H. Huo, A. Trewartha, E. A. Olivetti, G. Ceder, Opportunities and Challenges of Text Mining in Aterials Research, *iScience*, **2021**, *24*, 102155.

[3] H. Huo, C. J. Bartel, T. He, A. Trewartha, A. Dunn, B. Ouyang, A. Jain, G. Ceder, Machine-Learning Rationalization and Prediction of Solid-State Synthesis Conditions, *Chem. Mater.*, **2022**, *34*, 7323.

[4] V. Tshitoyan, J. Dagdelen, L. Weston, A. Dunn, Z. Rong, O. Kononova, K. A. Persson, G. Ceder, A. Jain, Unsupervised Word Embeddings Capture Latent Knowledge from Materials Science Literature, *Nature*, **2019**, *571*, 95.

[5] H. Huo, Z. Rong, O. Kononova, W. Sun, T. Botari, T. He, V. Tshitoyan, G. Ceder, Semi-Supervised Machine-Learning Classification of Materials Synthesis Procedures, *npj Computational Materials*, **2019**, *5*, 1.

[6] J. Dagdelen, A. Dunn, S. Lee, N. Walker, A. S. Rosen, G. Ceder, K. A. Persson, A. Jain, Structured Information Extraction from Scientific Text with Large Language Models, *Nat. Commun.*, **2024**, *15*, 1418.

[7] J. Devlin, M.-W. Chang, K. Lee, K. Toutanova, BERT: Pre-Training of Deep Bidirectional Transformers for Language Understanding, *arXiv [cs.CL]*, **2018**.

[8] T. B. Brown, B. Mann, N. Ryder, M. Subbiah, J. Kaplan, P. Dhariwal, A. Neelakantan, P. Shyam, G. Sastry, A. Askell, S. Agarwal, A. Herbert-Voss, G. Krueger, T. Henighan, R. Child, A. Ramesh, D. M. Ziegler, J. Wu, C. Winter, C. Hesse, M. Chen, E. Sigler, M. Litwin, S. Gray, B. Chess, J. Clark, C. Berner, S. McCandlish, A. Radford, I. Sutskever, D. Amodei, Language Models Are Few-Shot Learners, *arXiv [cs.CL]*, **2020**.

[9] Gemini Team, R. Anil, S. Borgeaud, J.-B. Alayrac, J. Yu, R. Soricut, J. Schalkwyk, A. M. Dai, A. Hauth, K. Millican, D. Silver, M. Johnson, I. Antonoglou, J. Schrittwieser, A. Glaese, J. Chen, E. Pitler, T. Lillicrap, A. Lazaridou, O. Firat, J. Molloy, M. Isard, P. R. Barham, T. Hennigan, B. Lee, F. Viola, M. Reynolds, Y. Xu, R. Doherty, E. Collins, C. Meyer, E. Rutherford, E. Moreira, K. Ayoub, M. Goel, J. Krawczyk, C. Du, E. Chi, H.-T. Cheng, E. Ni, P. Shah, P. Kane, B. Chan, M. Faruqui, A. Severyn, H. Lin, Y. Li, Y. Cheng, A. Ittycheriah, M. Mahdieh, M. Chen, P. Sun, D. Tran, S. Bagri, B. Lakshminarayanan, J. Liu, A. Orban, F. Güra, H. Zhou, X. Song, A. Boffy, H. Ganapathy, S. Zheng, H. Choe, Á. Weisz, T. Zhu, Y. Lu, S. Gopal, J. Kahn, M. Kula, J. Pitman, R. Shah, E. Taropa, M. Al Merey, M. Baeuml, Z. Chen, L. El Shafey, Y. Zhang, O. Sercinoglu, G. Tucker, E. Piqueras, M. Krikun, I. Barr, N. Savinov, I. Danihelka, B. Roelofs, A. White, A. Andreassen, T. von Glehn, L. Yagati, M. Kazemi, L. Gonzalez, M. Khalman, J. Sygnowski, A. Frechette, C. Smith, L. Culp, L. Proleev, Y. Luan, X. Chen, J. Lottes, N. Schucher, F. Lebron, A. Rrustemi, N. Clay, P. Crone, T. Kocisky, J. Zhao, B. Perz, D. Yu, H. Howard, A. Bloniarz, J. W. Rae, H. Lu, L. Sifre, M. Maggioni, F. Alcober, D. Garrette, M. Barnes, S. Thakoor, J. Austin, G. Barth-Maron, W. Wong, R. Joshi, R. Chaabouni, D. Fatiha, A. Ahuja, G. S. Tomar, E. Senter, M. Chadwick, I. Kornakov, N. Attaluri, I. Iturrate, R. Liu, Y. Li, S. Cogan, J. Chen, C. Jia, C. Gu, Q. Zhang, J. Grimstad, A. J. Hartman, X. Garcia, T. S. Pillai, J. Devlin, M. Laskin, D. de Las Casas, D. Valter, C. Tao, L. Blanco, A. P. Badia, D. Reitter, M. Chen, J. Brennan, C. Rivera, S. Brin, S. Iqbal, G. Surita, J. Labanowski, A. Rao, S. Winkler, E. Parisotto, Y. Gu, K. Olszewska, R. Addanki, A. Miech, A. Louis, D. Teplyashin, G. Brown, E. Catt, J. Balaguer, J. Xiang, P. Wang, Z. Ashwood, A. Briukhov, A. Webson, S. Ganapathy, S. Sanghavi, A. Kannan, M.-W. Chang, A. Stjerngren, J. Djolonga, Y. Sun, A. Bapna, M. Aitchison, P. Pejman, H. Michalewski, T. Yu, C. Wang, J. Love, J. Ahn, D. Bloxwich, K. Han, P. Humphreys, T. Sellam, J. Bradbury, V. Godbole, S. Samangooei, B. Damoc, A. Kaskasoli, S. M. R. Arnold, V. Vasudevan, S. Agrawal, J. Riesa, D. Lepikhin, R. Tanburn, S. Srinivasan, H. Lim, S. Hodkinson, P. Shyam, J. Ferret, S. Hand, A. Garg, T. Le Paine, J. Li, Y. Li, M. Giang, A. Neitz, Z. Abbas, S. York, M. Reid, E. Cole, A. Chowdhery, D. Das, D. Rogozińska, V. Nikolaev, P. Sprechmann, Z. Nado, L. Zilka, F. Prost, L. He, M. Monteiro, G. Mishra, C. Welty, J. Newlan, D. Jia, M. Allamanis, C. H. Hu, R. de Liedekerke, J. Gilmer, C. Saroufim, S. Rijhwani, S. Hou, D. Shrivastava, A. Baddepudi, A. Goldin, A. Ozturel, A. Cassirer, Y. Xu, D. Sohn, D. Sachan, R. K. Amplayo, C. Swanson, D. Petrova, S. Narayan, A. Guez, S. Brahma, J. Landon, M. Patel, R. Zhao, K. Villela, L. Wang, W. Jia, M. Rahtz, M. Giménez, L. Yeung, J. Keeling, P. Georgiev, D. Mincu, B. Wu, S. Haykal, R. Saputro, K. Vodrahalli, J. Qin, Z. Cankara, A. Sharma, N. Fernando, W. Hawkins, B. Neyshabur, S. Kim, A. Hutter, P. Agrawal, A. Castro-Ros, G. van den Driessche, T. Wang, F. Yang, S.-Y. Chang, P. Komarek, R. McIlroy, M. Lučić, G. Zhang, W. Farhan, M. Sharman, P. Natsev, P. Michel, Y. Bansal, S. Qiao, K. Cao, S. Shakeri, C. Butterfield, J. Chung, P. K. Rubenstein, S. Agrawal, A. Mensch, K. Soparkar, K. Lenc, T. Chung, A. Pope, L. Maggiore, J. Kay, P. Jhakra, S. Wang, J. Maynez, M. Phuong, T. Tobin, A. Tacchetti, M. Trebacz, K. Robinson, Y. Katariya, S. Riedel, P. Bailey, K. Xiao, N. Ghelani, L. Aroyo, A. Slone, N. Houlsby, X. Xiong, Z. Yang, E. Gribovskaya, J. Adler, M. Wirth, L. Lee, Music Li, T. Kagohara, J. Pavagadhi, S. Bridgers, A. Bortsova, S. Ghemawat, Z. Ahmed, T. Liu, R. Powell, V. Bolina, M. Iinuma, P. Zablotskaia, J. Besley, D.-W. Chung, T. Dozat, R. Comanescu, X. Si, J. Greer, G. Su, M. Polacek, R. L. Kaufman, S. Tokumine, H. Hu, E. Buchatskaya, Y. Miao, M. Elhawaty, A. Siddhant, N. Tomasev, J. Xing, C. Greer, H. Miller, S. Ashraf, A. Roy, Z. Zhang, A. Ma, A. Filos, M. Besta, R. Blevins, T. Klimenko, C.-K. Yeh, S. Changpinyo, J. Mu, O. Chang, M. Pajarskas, C. Muir, V. Cohen, C. Le Lan, K. Haridasan, A. Marathe, S. Hansen, S. Douglas, R. Samuel, M. Wang, S. Austin, C. Lan, J. Jiang, J. Chiu, J. A. Lorenzo, L. L. Sjösund, S. Cevey, Z. Gleicher, T. Avrahami, A. Boral, H. Srinivasan, V. Selo, R. May, K. Aisopos, L. Hussenot, L. B. Soares, K. Baumli, M. B. Chang, A. Recasens, B. Caine, A. Pritzel, F. Pavetic, F. Pardo, A. Gergely, J. Frye, V. Ramasesh, D. Horgan, K. Badola, N. Kassner, S. Roy, E. Dyer, V. C. Campos, A. Tomala, Y. Tang, D. El Badawy, E. White, B. Mustafa, O. Lang, A. Jindal, S. Vikram, Z. Gong, S. Caelles, R. Hemsley, G. Thornton, F. Feng, W. Stokowiec, C. Zheng, P. Thacker, Ç. Ünlü, Z. Zhang, M. Saleh, J. Svensson, M. Bileschi, P. Patil, A. Anand, R. Ring, K. Tsihlas, A. Vezer, M. Selvi, T. Shevlane, M. Rodriguez, T. Kwiatkowski, S. Daruki, K. Rong, A. Dafoe, N. FitzGerald, K. Gu-Lemberg, M. Khan, L. A. Hendricks, M. Pellat, V. Feinberg, J. Cobon-Kerr, T. Sainath, M. Rauh, S. H. Hashemi, R. Ives, Y. Hasson, E. Noland, Y. Cao, N. Byrd, L. Hou, Q. Wang, T. Sottiaux, M. Paganini, J.-B. Lespiau, A. Moufarek, S. Hassan, K. Shivakumar, J. van Amersfoort, A. Mandhane, P. Joshi, A. Goyal, M. Tung, A. Brock, H. Sheahan, V. Misra, C. Li, N. Rakićević, M. Dehghani, F. Liu, S. Mittal, J. Oh, S. Noury, E. Sezener, F. Huot, M. Lamm, N. De Cao, C. Chen, S. Mudgal, R. Stella, K. Brooks, G. Vasudevan, C. Liu, M. Chain, N. Melinkeri, A. Cohen, V. Wang, K. Seymore, S. Zubkov, R. Goel, S. Yue, S. Krishnakumaran, B. Albert, N. Hurley, M. Sano, A. Mohananey, J. Joughin, E. Filonov, T. Kępa, Y. Eldawy, J. Lim, R. Rishi, S. Badiezadegan, T. Bos, J. Chang, S. Jain, S. G. S. Padmanabhan, S. Puttagunta, K. Krishna, L. Baker, N. Kalb, V. Bedapudi, A. Kurzrok, S. Lei, A. Yu, O. Litvin, X. Zhou, Z. Wu, S. Sobell, A. Siciliano, A. Papir, R. Neale, J. Bragagnolo, T. Toor, T. Chen, V. Anklin, F. Wang, R. Feng, M. Gholami, K. Ling, L. Liu, J. Walter, H. Moghaddam, A. Kishore, J. Adamek, T. Mercado, J. Mallinson, S. Wandekar, S. Cagle, E. Ofek, G. Garrido, C. Lombriser, M. Mukha, B. Sun, H. R. Mohammad, J. Matak, Y. Qian, V. Peswani, P. Janus, Q. Yuan, L. Schelin, O. David, A. Garg, Y. He, O. Duzhyi, A. Älgmyr, T. Lottaz, Q. Li, V. Yadav, L. Xu, A. Chinien, R. Shivanna, A. Chuklin, J. Li, C. Spadine, T. Wolfe, K. Mohamed, S. Das, Z. Dai, K. He, D. von Dincklage, S. Upadhyay, A. Maurya, L. Chi, S. Krause, K. Salama, P. G. Rabinovitch, K. R. M. Pavan, A. Selvan, M. Dektiarev, G. Ghiasi, E. Guven, H. Gupta, B. Liu, D. Sharma, I. H. Shtacher, S. Paul, O. Akerlund, F.-X. Aubet, T. Huang, C. Zhu, E. Zhu, E. Teixeira, M. Fritze, F. Bertolini, L.-E. Marinescu, M. Bölle, D. Paulus, K. Gupta, T. Latkar, M. Chang, J. Sanders, R. Wilson, X. Wu, Y.-X. Tan, L. N. Thiet, T. Doshi, S. Lall, S. Mishra, W. Chen, T. Luong, S. Benjamin, J. Lee, E. Andrejczuk, D. Rabiej, V. Ranjan, K. Styrc, P. Yin, J. Simon, M. R. Harriott, M. Bansal, A. Robsky, G. Bacon, D. Greene, D. Mirylenka, C. Zhou, O. Sarvana, A. Goyal, S. Andermatt, P. Siegler, B. Horn, A. Israel, F. Pongetti, C.-W. “louis” Chen, M. Selvatici, P. Silva, K. Wang, J. Tolins, K. Guu, R. Yogev, X. Cai, A. Agostini, M. Shah, H. Nguyen, N. Ó. Donnaile, S. Pereira, L. Friso, A. Stambler, A. Kurzrok, C. Kuang, Y. Romanikhin, M. Geller, Z. J. Yan, K. Jang, C.-C. Lee, W. Fica, E. Malmi, Q. Tan, D. Banica, D. Balle, R. Pham, Y. Huang, D. Avram, H. Shi, J. Singh, C. Hidey, N. Ahuja, P. Saxena, D. Dooley, S. P. Potharaju, E. O’Neill, A. Gokulchandran, R. Foley, K. Zhao, M. Dusenberry, Y. Liu, P. Mehta, R. Kotikalapudi, C. Safranek-Shrader, A. Goodman, J. Kessinger, E. Globen, P. Kolhar, C. Gorgolewski, A. Ibrahim, Y. Song, A. Eichenbaum, T. Brovelli, S. Potluri, P. Lahoti, C. Baetu, A. Ghorbani, C. Chen, A. Crawford, S. Pal, M. Sridhar, P. Gurita, A. Mujika, I. Petrovski, P.-L. Cedoz, C. Li, S. Chen, N. D. Santo, S. Goyal, J. Punjabi, K. Kappaganthu, C. Kwak, L. V. Pallavi, S. Velury, H. Choudhury, J. Hall, P. Shah, R. Figueira, M. Thomas, M. Lu, T. Zhou, C. Kumar, T. Jurdi, S. Chikkerur, Y. Ma, A. Yu, S. Kwak, V. Ähdel, S. Rajayogam, T. Choma, F. Liu, A. Barua, C. Ji, J. H. Park, V. Hellendoorn, A. Bailey, T. Bilal, H. Zhou, M. Khatir, C. Sutton, W. Rzadkowski, F. Macintosh, K. Shagin, P. Medina, C. Liang, J. Zhou, P. Shah, Y. Bi, A. Dankovics, S. Banga, S. Lehmann, M. Bredesen, Z. Lin, J. E. Hoffmann, J. Lai, R. Chung, K. Yang, N. Balani, A. Bražinskas, A. Sozanschi, M. Hayes, H. F. Alcalde, P. Makarov, W. Chen, A. Stella, L. Snijders, M. Mandl, A. Kärrman, P. Nowak, X. Wu, A. Dyck, K. Vaidyanathan, R. Raghavender, J. Mallet, M. Rudominer, E. Johnston, S. Mittal, A. Udathu, J. Christensen, V. Verma, Z. Irving, A. Santucci, G. Elsayed, E. Davoodi, M. Georgiev, I. Tenney, N. Hua, G. Cideron, E. Leurent, M. Alnahlawi, I. Georgescu, N. Wei, I. Zheng, D. Scandinaro, H. Jiang, J. Snoek, M. Sundararajan, X. Wang, Z. Ontiveros, I. Karo, J. Cole, V. Rajashekhar, L. Tumeh, E. Ben-David, R. Jain, J. Uesato, R. Datta, O. Bunyan, S. Wu, J. Zhang, P. Stanczyk, Y. Zhang, D. Steiner, S. Naskar, M. Azzam, M. Johnson, A. Paszke, C.-C. Chiu, J. S. Elias, A. Mohiuddin, F. Muhammad, J. Miao, A. Lee, N. Vieillard, J. Park, J. Zhang, J. Stanway, D. Garmon, A. Karmarkar, Z. Dong, J. Lee, A. Kumar, L. Zhou, J. Evens, W. Isaac, G. Irving, E. Loper, M. Fink, I. Arkatkar, N. Chen, I. Shafran, I. Petrychenko, Z. Chen, J. Jia, A. Levskaya, Z. Zhu, P. Grabowski, Y. Mao, A. Magni, K. Yao, J. Snaider, N. Casagrande, E. Palmer, P. Suganthan, A. Castaño, I. Giannoumis, W. Kim, M. Rybiński, A. Sreevatsa, J. Prendki, D. Soergel, A. Goedeckemeyer, W. Gierke, M. Jafari, M. Gaba, J. Wiesner, D. G. Wright, Y. Wei, H. Vashisht, Y. Kulizhskaya, J. Hoover, M. Le, L. Li, C. Iwuanyanwu, L. Liu, K. Ramirez, A. Khorlin, A. Cui, L. I. N. Tian, M. Wu, R. Aguilar, K. Pallo, A. Chakladar, G. Perng, E. A. Abellan, M. Zhang, I. Dasgupta, N. Kushman, I. Penchev, A. Repina, X. Wu, T. van der Weide, P. Ponnapalli, C. Kaplan, J. Simsa, S. Li, O. Dousse, F. Yang, J. Piper, N. Ie, R. Pasumarthi, N. Lintz, A. Vijayakumar, D. Andor, P. Valenzuela, M. Lui, C. Paduraru, D. Peng, K. Lee, S. Zhang, S. Greene, D. D. Nguyen, P. Kurylowicz, C. Hardin, L. Dixon, L. Janzer, K. Choo, Z. Feng, B. Zhang, A. Singhal, D. Du, D. McKinnon, N. Antropova, T. Bolukbasi, O. Keller, D. Reid, D. Finchelstein, M. A. Raad, R. Crocker, P. Hawkins, R. Dadashi, C. Gaffney, K. Franko, A. Bulanova, R. Leblond, S. Chung, H. Askham, L. C. Cobo, K. Xu, F. Fischer, J. Xu, C. Sorokin, C. Alberti, C.-C. Lin, C. Evans, A. Dimitriev, H. Forbes, D. Banarse, Z. Tung, M. Omernick, C. Bishop, R. Sterneck, R. Jain, J. Xia, E. Amid, F. Piccinno, X. Wang, P. Banzal, D. J. Mankowitz, A. Polozov, V. Krakovna, S. Brown, M. Bateni, D. Duan, V. Firoiu, M. Thotakuri, T. Natan, M. Geist, S. T. Girgin, H. Li, J. Ye, O. Roval, R. Tojo, M. Kwong, J. Lee-Thorp, C. Yew, D. Sinopalnikov, S. Ramos, J. Mellor, A. Sharma, K. Wu, D. Miller, N. Sonnerat, D. Vnukov, R. Greig, J. Beattie, E. Caveness, L. Bai, J. Eisenschlos, A. Korchemniy, T. Tsai, M. Jasarevic, W. Kong, P. Dao, Z. Zheng, F. Liu, F. Yang, R. Zhu, T. H. Teh, J. Sanmiya, E. Gladchenko, N. Trdin, D. Toyama, E. Rosen, S. Tavakkol, L. Xue, C. Elkind, O. Woodman, J. Carpenter, G. Papamakarios, R. Kemp, S. Kafle, T. Grunina, R. Sinha, A. Talbert, D. Wu, D. Owusu-Afriyie, C. Du, C. Thornton, J. Pont-Tuset, P. Narayana, J. Li, S. Fatehi, J. Wieting, O. Ajmeri, B. Uria, Y. Ko, L. Knight, A. Héliou, N. Niu, S. Gu, C. Pang, Y. Li, N. Levine, A. Stolovich, R. Santamaria-Fernandez, S. Goenka, W. Yustalim, R. Strudel, A. Elqursh, C. Deck, H. Lee, Z. Li, K. Levin, R. Hoffmann, D. Holtmann-Rice, O. Bachem, S. Arora, C. Koh, S. H. Yeganeh, S. Põder, M. Tariq, Y. Sun, L. Ionita, M. Seyedhosseini, P. Tafti, Z. Liu, A. Gulati, J. Liu, X. Ye, B. Chrzaszcz, L. Wang, N. Sethi, T. Li, B. Brown, S. Singh, W. Fan, A. Parisi, J. Stanton, V. Koverkathu, C. A. Choquette-Choo, Y. Li, T. J. Lu, A. Ittycheriah, P. Shroff, M. Varadarajan, S. Bahargam, R. Willoughby, D. Gaddy, G. Desjardins, M. Cornero, B. Robenek, B. Mittal, B. Albrecht, A. Shenoy, F. Moiseev, H. Jacobsson, A. Ghaffarkhah, M. Rivière, A. Walton, C. Crepy, A. Parrish, Z. Zhou, C. Farabet, C. Radebaugh, P. Srinivasan, C. van der Salm, A. Fidjeland, S. Scellato, E. Latorre-Chimoto, H. Klimczak-Plucińska, D. Bridson, D. de Cesare, T. Hudson, P. Mendolicchio, L. Walker, A. Morris, M. Mauger, A. Guseynov, A. Reid, S. Odoom, L. Loher, V. Cotruta, M. Yenugula, D. Grewe, A. Petrushkina, T. Duerig, A. Sanchez, S. Yadlowsky, A. Shen, A. Globerson, L. Webb, S. Dua, D. Li, S. Bhupatiraju, D. Hurt, H. Qureshi, A. Agarwal, T. Shani, M. Eyal, A. Khare, S. R. Belle, L. Wang, C. Tekur, M. S. Kale, J. Wei, R. Sang, B. Saeta, T. Liechty, Y. Sun, Y. Zhao, S. Lee, P. Nayak, D. Fritz, M. R. Vuyyuru, J. Aslanides, N. Vyas, M. Wicke, X. Ma, E. Eltyshev, N. Martin, H. Cate, J. Manyika, K. Amiri, Y. Kim, X. Xiong, K. Kang, F. Luisier, N. Tripuraneni, D. Madras, M. Guo, A. Waters, O. Wang, J. Ainslie, J. Baldridge, H. Zhang, G. Pruthi, J. Bauer, F. Yang, R. Mansour, J. Gelman, Y. Xu, G. Polovets, J. Liu, H. Cai, W. Chen, X. Sheng, E. Xue, S. Ozair, C. Angermueller, X. Li, A. Sinha, W. Wang, J. Wiesinger, E. Koukoumidis, Y. Tian, A. Iyer, M. Gurumurthy, M. Goldenson, P. Shah, M. K. Blake, H. Yu, A. Urbanowicz, J. Palomaki, C. Fernando, K. Durden, H. Mehta, N. Momchev, E. Rahimtoroghi, M. Georgaki, A. Raul, S. Ruder, M. Redshaw, J. Lee, D. Zhou, K. Jalan, D. Li, B. Hechtman, P. Schuh, M. Nasr, K. Milan, V. Mikulik, J. Franco, T. Green, N. Nguyen, J. Kelley, A. Mahendru, A. Hu, J. Howland, B. Vargas, J. Hui, K. Bansal, V. Rao, R. Ghiya, E. Wang, K. Ye, J. M. Sarr, M. M. Preston, M. Elish, S. Li, A. Kaku, J. Gupta, I. Pasupat, D.-C. Juan, M. Someswar, T. M., X. Chen, A. Amini, A. Fabrikant, E. Chu, X. Dong, A. Muthal, S. Buthpitiya, S. Jauhari, N. Hua, U. Khandelwal, A. Hitron, J. Ren, L. Rinaldi, S. Drath, A. Dabush, N.-J. Jiang, H. Godhia, U. Sachs, A. Chen, Y. Fan, H. Taitelbaum, H. Noga, Z. Dai, J. Wang, C. Liang, J. Hamer, C.-S. Ferng, C. Elkind, A. Atias, P. Lee, V. Listík, M. Carlen, J. van de Kerkhof, M. Pikus, K. Zaher, P. Müller, S. Zykova, R. Stefanec, V. Gatsko, C. Hirnschall, A. Sethi, X. F. Xu, C. Ahuja, B. Tsai, A. Stefanoiu, B. Feng, K. Dhandhania, M. Katyal, A. Gupta, A. Parulekar, D. Pitta, J. Zhao, V. Bhatia, Y. Bhavnani, O. Alhadlaq, X. Li, P. Danenberg, D. Tu, A. Pine, V. Filippova, A. Ghosh, B. Limonchik, B. Urala, C. K. Lanka, D. Clive, Y. Sun, E. Li, H. Wu, K. Hongtongsak, I. Li, K. Thakkar, K. Omarov, K. Majmundar, M. Alverson, M. Kucharski, M. Patel, M. Jain, M. Zabelin, P. Pelagatti, R. Kohli, S. Kumar, J. Kim, S. Sankar, V. Shah, L. Ramachandruni, X. Zeng, B. Bariach, L. Weidinger, T. Vu, A. Subramanya, S. Hsiao, D. Hassabis, K. Kavukcuoglu, A. Sadovsky, Q. Le, T. Strohman, Y. Wu, S. Petrov, J. Dean, O. Vinyals, Gemini: A Family of Highly Capable Multimodal Models, *arXiv [cs.CL]*, **2023**.

[10] H. Touvron, T. Lavril, G. Izacard, X. Martinet, M.-A. Lachaux, T. Lacroix, B. Rozière, N. Goyal, E. Hambro, F. Azhar, A. Rodriguez, A. Joulin, E. Grave, G. Lample, LLaMA: Open and Efficient Foundation Language Models, *arXiv [cs.CL]*, **2023**.

[11] Z. Ji, N. Lee, R. Frieske, T. Yu, D. Su, Y. Xu, E. Ishii, Y. Bang, A. Madotto, P. Fung, Survey of Hallucination in Natural Language Generation, *ACM Comput. Surv.*, **2022**, DOI 10.1145/3571730.

[12] A. C. Ferrari, D. M. Basko, Raman Spectroscopy as a Versatile Tool for Studying the Properties of Graphene, *Nat. Nanotechnol.*, **2013**, *8*, 235.

[13] X. Cong, X.-L. Liu, M.-L. Lin, P.-H. Tan, Application of Raman Spectroscopy to Probe Fundamental Properties of Two-Dimensional Materials, *npj 2D Materials and Applications*, **2020**, *4*, 1.

[14] F. Liang, H. Xu, X. Wu, C. Wang, C. Luo, J. Zhang, Raman Spectroscopy Characterization of Two-Dimensional Materials*, *Chin. Physics B*, **2018**, *27*, 037802.

[15] A. Taghizadeh, U. Leffers, T. G. Pedersen, K. S. Thygesen, A Library of Ab Initio Raman Spectra for Automated Identification of 2D Materials, *Nat. Commun.*, **2020**, *11*, 3011.

[16] T. Gupta, M. Zaki, N. M. A. Krishnan, Mausam, MatSciBERT: A Materials Domain Language Model for Text Mining and Information Extraction, *npj Computational Materials*, **2022**, *8*, 1.

[17] A. Otegi, J. A. Campos, G. Azkune, A. Soroa, E. Agirre, in *Proceedings of the 1st Workshop on NLP for COVID-19 (Part 2) at EMNLP 2020*, Association For Computational Linguistics, Online, **2020**.

[18] “Question Answering in Context,” can be found under https://quac.ai/, **n.d.**

[19] I. H. Abidi, S. P. Giridhar, J. O. Tollerud, J. Limb, M. Waqar, A. Mazumder, E. L. H. Mayes, B. J. Murdoch, C. Xu, A. Bhoriya, A. Ranjan, T. Ahmed, Y. Li, J. A. Davis, C. L. Bentley, S. P. Russo, E. D. Gaspera, S. Walia, Oxygen Driven Defect Engineering of Monolayer MoS_2_ for Tunable Electronic, Optoelectronic, and Electrochemical Devices, *Adv. Funct. Mater.*, **2024**, *34*, 2402402.

[20] H. K. Paidi, R. Mudunuri, D. J. Babu, Exploring MoS2 Growth: A Comparative Study of Atmospheric and Low-Pressure CVD, *Langmuir*, **2024**, *40*, 25648.

[21] I. A. Moses, C. Chen, J. M. Redwing, W. F. Reinhart, Cross‐modal Characterization of Thin‐film MoS_2_ Using Generative Models, *Adv. Intell. Syst.*, **2025**, 2500613.

[22] S. Huang, J. M. Cole, BatteryBERT: A Pretrained Language Model for Battery Database Enhancement, *J. Chem. Inf. Model.*, **2022**, *62*, 6365.
